# Deep Learning in Proteomics

**DOI:** 10.1002/pmic.201900335

**Published:** 2020-10-30

**Authors:** Bo Wen, Wen‐Feng Zeng, Yuxing Liao, Zhiao Shi, Sara R. Savage, Wen Jiang, Bing Zhang

**Affiliations:** ^1^ Lester and Sue Smith Breast Center Baylor College of Medicine Houston TX 77030 USA; ^2^ Department of Molecular and Human Genetics Baylor College of Medicine Houston TX 77030 USA; ^3^ Key Lab of Intelligent Information Processing of Chinese Academy of Sciences (CAS) Chinese Academy of Sciences Institute of Computing Technology Beijing 100190 China

**Keywords:** bioinformatics, deep learning, proteomics

## Abstract

Proteomics, the study of all the proteins in biological systems, is becoming a data‐rich science. Protein sequences and structures are comprehensively catalogued in online databases. With recent advancements in tandem mass spectrometry (MS) technology, protein expression and post‐translational modifications (PTMs) can be studied in a variety of biological systems at the global scale. Sophisticated computational algorithms are needed to translate the vast amount of data into novel biological insights. Deep learning automatically extracts data representations at high levels of abstraction from data, and it thrives in data‐rich scientific research domains. Here, a comprehensive overview of deep learning applications in proteomics, including retention time prediction, MS/MS spectrum prediction, de novo peptide sequencing, PTM prediction, major histocompatibility complex‐peptide binding prediction, and protein structure prediction, is provided. Limitations and the future directions of deep learning in proteomics are also discussed. This review will provide readers an overview of deep learning and how it can be used to analyze proteomics data.

## 1 Introduction

Mass spectrometry (MS) has been widely used for both untargeted and targeted proteomics studies. For untargeted proteomics, all proteins extracted from a sample are digested into peptides and then injected into a liquid chromatography‐tandem mass spectrometry (LC‐MS/MS) system for detection using the data‐dependent acquisition (DDA) method or the data‐independent acquisition (DIA) method. In contrast, targeted proteomics only detects selected proteins of interest using the multiple reaction monitoring (MRM) method (also known as selected reaction monitoring) or parallel reaction monitoring (PRM) method. With advancements of both LC and MS technologies in recent years, large volumes of MS/MS data have been generated. A typical DDA or DIA experiment can produce hundreds of thousands of MS/MS spectra. Sophisticated algorithms and tools are required for raw data processing, data quality control, peptide and protein identification and quantification, post‐translational modification (PTM) detection, and downstream analyses. Due to these computational requirements, machine learning methods have been widely used in many aspects of proteomics data analysis.^[^ ^1^, ^2^, ^3^ ^]^

Deep learning is a sub‐discipline of machine learning. It has advanced rapidly during the last two decades and has demonstrated superior performance in various fields including computer vision, speech recognition, natural‐language processing, bioinformatics, and medical image analysis. Deep learning is based on artificial neural networks with representation learning that aim to mimic the human brain. The key difference between deep learning and traditional machine learning algorithms such as support vector machine (SVM) and random forests (RF) is that deep learning can automatically learn features and patterns from data without handcrafted feature engineering. Therefore, deep learning is particularly suited to scientific domains where large, complex datasets are available.

Deep learning has already been applied to various aspects of biological research, including analyses of medical image data, gene expression data, DNA and protein sequence data.^[^ ^4^ ^]^ A number of reviews have been published to provide an overview of deep learning applications in biomedicine,^[^ ^5^ ^]^ clinical diagnostics,^[^ ^6^ ^]^ bioinformatics,^[^ ^7^ ^]^ and genomics.^[^ ^8^ ^]^

**Figure 1:**
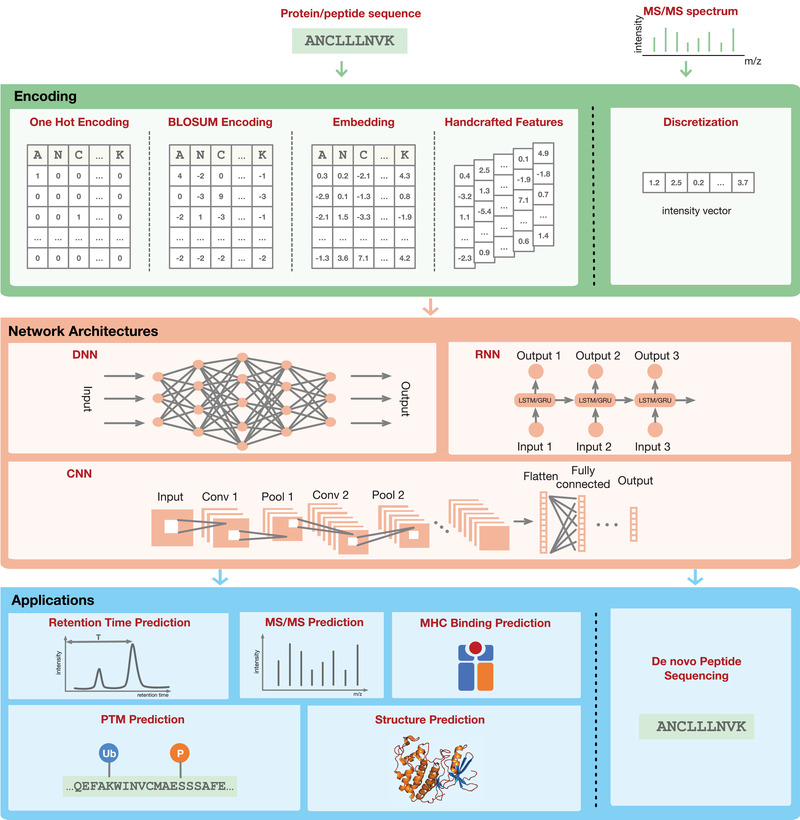
Overview of the key components of deep learning and its applications in proteomics.

The aim of this paper is to provide the proteomics community a comprehensive overview of deep learning applications for the analysis of proteomics data. We first introduce fundamental concepts in deep learning. We then present a survey of major applications including retention time (RT) prediction, MS/MS spectrum prediction, de novo peptide sequencing, PTM prediction, major histocompatibility complex (MHC)‐peptide binding prediction, and protein structure prediction (**Figure** **1**). Finally, we discuss future directions and limitations of deep learning in proteomics.

## 2 Basic Concepts in Deep Learning

Deep learning seeks to learn the representation of data through a series of successive layers of increasing abstraction.^[^ ^9^ ^]^ These layered representations are learned via models called artificial neural networks (ANNs). ANNs, where many simple units called neurons are connected to each another with different weights, simulate the mechanism of learning in the human brain. These weights serve the same role as the indication of strengths between synaptic connections in biological organisms. Training a neural network requires the following components: training samples with input data (e.g., peptide sequences) and matching targets (e.g., retention times of the peptides), a network model, a loss function, and an optimization method. The network model, with multiple layers connected together, maps the input data to predictions. A loss function then computes a loss value which measures how well the network's predictions match the expected outcomes by comparing these predictions with the targets. The optimization method uses this loss value as a feedback signal to incrementally adjust the weights of the network connections in order to optimize the model. This method of finding optimal weights for the neural network is called backpropagation.^[^ ^9^ ^]^ The target variable can be categorical or continuous. Whereas the former corresponds to classification problems, the later corresponds to regression problems.

One important aspect of deep learning, or machine learning in general, is data preprocessing or input encoding to make raw data, such as peptide or protein sequences, more amenable to the models. Typically, all input and output variables are required to be numeric. MS/MS spectra can be simply discretized to produce an intensity vector.^[^ ^10^ ^]^ For sequence‐based data such as peptide and protein sequences, the sequence is first segmented into tokens (amino acids) and then each token is associated with a numeric vector. There are multiple ways to associate a vector with a token (Figure 1). One of the simplest and most widely used methods is called one‐hot encoding where each amino acid is represented by a unit binary vector of length *n*, containing a single one and *n*‐1 zeros (e.g., [1,0,0, …, 0] for one amino acid and [0,1,0, …, 0] for another amino acid). This solution treats all amino acids equally without using any prior knowledge. Another approach is to use the BLOcks SUbstitution Matrix (BLOSUM) for encoding, representing each amino acid by its corresponding row in the BLOSUM matrix.^[^ ^11^ ^]^ Instead of treating all amino acids independently, the BLOSUM matrix derived from protein sequence alignments keeps the evolutionary information about which pairs of amino acids are easily interchangeable during evolution. This information may be useful in certain applications such as MHC‐peptide binding prediction. Another way to encode amino acid sequences is the use of dense numeric vectors, also called word embedding, which is widely used in natural language processing.^[^ ^12^ ^]^ Unlike the sparse vectors obtained via one‐hot encoding where most elements are zero, these vectors could be learned from large unlabeled protein datasets, such as all sequences pulled from the UniProt database, in an unsupervised manner.^[^ ^13^ ^]^ These vectors could also be learned jointly with the main task (e.g., RT prediction or MHC‐peptide binding prediction) in the same way that the weights of the neural network of the main task are learned.^[^ ^14^ ^]^ This type of encoding method has been demonstrated to be extremely useful in certain tasks.^[^ ^12^, ^14^, ^15^, ^16^ ^]^ Before encoding a sequence as dense numeric vectors, the sequence is typically represented as an integer vector in which each token is represented by a unique integer. The final method is to design handcrafted features and then take these features as input for modeling. This is the most common method used in traditional machine learning and is different from the previous three methods, in which handcrafted feature engineering is typically not required.

The behavior of neural networks is largely shaped by its network architecture. A network's architecture can generally be characterized by: 1) number of neurons in each layer, 2) number of layers, and 3) types of connections between layers. The most well‐known architectures include: deep neural networks (DNNs), convolutional neural networks (CNNs), and recurrent neural networks (RNNs) (Figure 1). In this review, DNNs refer to networks that consist of an input layer, multiple hidden layers and an output layer. Nodes from adjacent layers are fully connected with each other. CNNs mainly consist of convolutional layers and pooling layers, frequently followed by a number of fully connected layers. One of the key processes of CNNs is to slide a filter over the input (such as an image or a sequence), where different filters can capture different patterns in the input data. CNNs have been widely used in the analysis of medical image data and have also been applied to DNA and protein sequence data.^[^ ^4^ ^]^ Unlike CNNs, RNNs process an input sequence one element at a time step by using recurrent and cyclic connection units, and the output for each step depends not only on the current element but also on previous elements. RNNs can capture long‐range interactions within the sequence and are well‐suited to model sequential data such as DNA or protein sequences. For example, if an input sequence is a peptide or protein sequence, each element could be an amino acid.

Conventional RNNs typically suffer from what are called the vanishing and exploding gradient problems when the sequence is very long.^[^ ^17^ ^]^ Although it is theoretically capable of retaining the information about inputs seen many time steps earlier at time step *t*, in practice, such long‐term dependencies are difficult to learn. This happens when the gradients used to update the weights become extremely small or large and do not contribute to the learning process or render the model too unstable for continued learning. In other words, the RNNs become untrainable. To overcome this, novel network architectures such as long short‐term memory units (LSTMs)^[^ ^18^ ^]^ and gated recurrent units (GRUs)^[^ ^19^ ^]^ were proposed. They have internal mechanisms called gates that can regulate the flow of information. These gates can learn which data in a sequence is important to keep or discard, thus preventing older signals from gradually vanishing or exploding during processing. To allow RNNs to have both backward and forward information about the sequence at every time step, two independent RNNs can be used together to form a new network called bidirectional RNN (BiRNN). The input sequence is fed in normal order for one RNN, and in reverse order for the other one. The outputs of the two RNNs are then concatenated at each step. If LSTMs or GRUs are employed, it is then called bidirectional long short‐term memory (BiLSTM) or bidirectional gated recurrent unit (BiGRU), respectively.

Other newer network architectures are being continuously developed. For example, capsule networks (CapsNets)^[^ ^20^ ^]^ group neurons in each layer into multiple capsules, allowing better modeling of hierarchical relationships inside a neural network. A deep learning algorithm may also combine different types of architectures in a network. For example, combining CNN and RNN (LSTM or GRU) in one network could leverage the strengths of both architectures to achieve better performance than just using one of them.

Deep learning has already been used in a number of proteomics applications (Figure 1), in which the overall workflow described above is generally applicable. However, individual tasks may require additional customization.

## 3 Deep Learning for Retention Time Prediction

In MS‐based proteomics experiments, peptide mixtures are typically separated via an LC system prior to analysis by MS. The retention time of a peptide refers to the time point when the peptide elutes from the LC column in an LC‐MS/MS system, which is recorded by the instrument. Retention time of a peptide is determined by the degree of the peptide interaction with the stationary and mobile phases of the LC system. The retention time of peptides is highly reproducible under the same LC conditions. Accurately predicted retention times have several applications in MS‐based proteomics, including 1) improving sensitivity of peptide identification in database searching,^[^ ^21^, ^22^, ^23^, ^24^ ^]^ 2) serving as a quality evaluation metric for peptide identification,^[^ ^25^, ^26^, ^27^, ^28^ ^]^ 3) building spectral libraries for DIA data analysis,^[^ ^29^, ^30^, ^31^, ^32^, ^33^ ^]^ and 4) facilitating targeted proteomics experiments.

Studies of peptide RT prediction can be tracked back to the 1980s^[^ ^34^, ^35^ ^]^ with studies continuing to focus on improving RT prediction to this day.^[^ ^21^, ^25^, ^29^, ^36^, ^37^, ^38^, ^39^ ^]^ Methods for peptide RT prediction can be divided into two primary categories: index‐based methods, such as SSRCalc,^[^ ^40^, ^41^ ^]^ and machine learning‐based methods. Machine learning‐based methods can be further divided into two sub groups: traditional machine learning‐based methods including Elude^[^ ^42^, ^43^ ^]^ and GPTime,^[^ ^38^ ^]^ and deep learning‐based methods including DeepRT,^[^ ^44^ ^]^ Prosit,^[^ ^29^ ^]^ DeepMass,^[^ ^45^ ^]^ Guan et al.,^[^ ^46^ ^]^ DeepDIA,^[^ ^30^ ^]^ AutoRT,^[^ ^25^ ^]^ and DeepLC.^[^ ^47^ ^]^ As shown in **Table** **1**, deep learning‐based tools can be divided into three groups based on the type of neural network architecture used: RNN‐based, CNN‐based, and hybrid networks, with RNN as the dominant architecture because it was developed for sequential data modeling. Several of these tools also have a separate module for MS/MS spectrum prediction (see next Section).

**Table 1:** List of deep learning‐based retention time prediction tools

No.	Software	Framework	Core network model	Input encoding	Usability^a)^	Year	Reference
1	DeepRT	PyTorch	CNN	Word embedding	O,C,P,T	2018	[44]
2	Prosit	Keras/TensorFlow	RNN	Word embedding	O,C,W,P,T	2019	[29]
3	DeepMass	Keras/TensorFlow	RNN	One‐hot	‐	2019	[45]
4	Guan et al.	Keras/TensorFlow	RNN	One‐hot	O,C,P,T	2019	[46]
5	DeepDIA	Keras/TensorFlow	CNN+RNN	One‐hot	O,C,P,T	2020	[30]
6	AutoRT	Keras/TensorFlow	CNN+RNN	One‐hot	O,C,P,T	2020	[25]
7	DeepLC	TensorFlow	CNN	One‐hot, global features, amino/diamino acids composition	O,G,C,P,T	2020	[47]

^a)^ O, open‐source; G, graphical user interface; C, command line; P, provide trained model for prediction; W, web interface; T, provide option for model training. The link of each tool could be found at https://github.com/bzhanglab/deep_learning_in_proteomics.

Prosit is a representative tool of the RNN‐based group. In Prosit, a peptide sequence is represented as a discrete integer vector of length 30, with each non‐zero integer mapping to one amino acid and padded with zeros for sequences shorter than 30 amino acids. The padding operation forces all encoded peptides to have the same length. The deep neural network for RT prediction in Prosit consists of an encoder and a decoder. The encoder encodes the input peptide sequence data into a latent representation, whereas the decoder decodes the representation to predict RT. The peptide encoder consists of an embedding layer, a BiGRU layer, a recurrent GRU layer, and an attention layer.^[^ ^48^ ^]^ The learned representation of the input peptides captures the intrinsic relations of different amino acids. The decoder connects the latent representation learned from the encoder to a dense layer to make predictions. Prosit was shown to outperform SSRCalc and Elude for RT prediction in the original study.^[^ ^29^ ^]^ The RT prediction method proposed in DeepMass is also based on RNN architecture. DeepMass uses one‐hot encoding for peptide sequence representation, and the network includes a BiLSTM layer and another LSTM layer followed by two dense layers. DeepMass was compared to SSRCalc in the original study and showed superior performance.^[^ ^45^ ^]^ The RT model proposed by Guan et al.^[^ ^46^ ^]^ is similar to DeepMass; however, it uses two BiLSTM layers, and a masking layer is used to discard padding sequences during training and prediction.

Both DeepRT and DeepLC use CNN‐based architectures, with DeepRT specifically using a CapsNet which is a variant of CNN. Similar to Prosit, DeepRT includes an embedding layer as the first layer of the neural network. In contrast, DeepLC uses a standard CNN framework. A unique feature of DeepLC, compared with all other tools in Table 1, is the ability to predict RT for peptides with modifications that are not present in the training data. This is mainly achieved by using a new peptide encoding based on atomic composition. Specifically, each peptide is encoded as a matrix with a dimension of 60 for the peptide sequence by 6 for the atom counts (C, H, N, O, P, and S). For a peptide with a length shorter than 60 amino acids, it will be padded with the character “X” without atomic composition to make it the same length of 60. For modified amino acids, the atomic composition of the modification is added to the atomic composition of the unmodified residue. In addition to this encoding, a peptide is further encoded in three additional ways to capture other position‐specific information and global information. The four encoding results are fed into the network through different paths. The last part of the network consists of six connected dense layers, which take as input the outputs from the previous paths. DeepLC showed comparable performance to the state‐of‐the‐art RT prediction algorithms for unmodified peptides and achieved similar performance for unseen modified peptides to that for the unmodified peptides.^[^ ^47^ ^]^

Other RT prediction models, such as DeepDIA and a model we developed called AutoRT, combine both CNN and RNN in the same networks. In DeepDIA, one‐hot encoded peptide sequences are fed into a CNN network, which is followed by a BiLSTM network. AutoRT uses a similar strategy to combine CNN and RNN networks, but GRU rather than LSTM is used. One unique feature of AutoRT is the use of a genetic algorithm to enable automatic deep neural network architecture search (NAS), through which the ten best‐performing models are identified and ensembled for RT prediction. NAS is a fast growing research area. The architectures from NAS have been demonstrated to be on par with or outperform hand‐designed architectures in many tasks.^[^ ^49^, ^50^ ^]^ Another feature of AutoRT is the use of transfer learning. Specifically, base models are trained using a large public dataset (>100 000 peptides), and then the trained base models are fine‐tuned using data from an experiment of interest to develop experiment‐specific models. By leveraging large public datasets, transfer learning makes it possible to obtain a highly accurate model even with a small size of experiment‐specific training data (≈700 peptides). This is very useful because only a few thousand peptides may be identified in a single run in many experiments.^[^ ^25^ ^]^

Accurate RT predictions from deep learning models have led to promising applications. For example, we used the difference (ΔRT) between AutoRT predicted RT and experimentally observed RT for each identified peptide as an evaluation metric for comparing different quality control strategies for variant peptide identification.^[^ ^25^ ^]^ The evaluation results provide insights and practical guidance on the selection of quality control strategies for variant peptide identification. Similarly, Li et al.^[^ ^51^ ^]^ used ΔRT derived from AutoRT prediction as a feature to rescore peptide spectrum matches (PSMs) in the analysis of immunopeptidomics data. Interestingly, rescoring with AutoRT led to significantly improved sensitivity of peptide identification, while rescoring with the ΔRT feature derived from the traditional machine learning‐based tool GPTime only showed minor improvement.^[^ ^51^ ^]^ Deep learning‐based RT prediction can also be used together with MS/MS spectrum prediction to build an in silico spectral library for DIA data analysis, as demonstrated in a few recent studies.^[^ ^30^, ^31^, ^32^ ^]^ Deep learning‐based RT prediction has not been used in any published targeted proteomics studies, but we expect this to change in the near future.

Although significant improvement has been made for peptide RT prediction using deep learning, RT prediction for peptides with modifications remains a major challenge. Some existing models consider a few common artifactual modifications, such as oxidation of methionine.^[^ ^25^, ^46^ ^]^ In these models, modified and unmodified amino acids are processed equally. These models can be used to predict RT for peptides containing these modifications, but the prediction errors are likely to be higher than those for peptides without modification due to the relative low frequency of modified amino acids in the training data. DeepLC is the only model that can predict RT for peptides containing modifications not present in the training data. However, the performance of RT prediction for the modifications that are chemically very different from anything encountered in the training set, such as phosphorylation, is obviously lower than others. Moreover, peptide encoding considering atomic composition cannot differentiate between isomeric structures that are physicochemically different. RT prediction for peptides with complicated modifications such as glycosylation is even more difficult. There is no deep learning‐based tool reported to predict RTs for intact glycosylated peptides yet. Thus, new training strategies or deep learning networks are needed to improve RT prediction for peptides with modifications. Moreover, all existing deep learning‐based tools are developed for RT prediction of linear peptides. They cannot be used for RT prediction for cross‐linked peptides generated using cross‐linking mass spectrometry, in which two peptides are typically connected to form a cross‐linked peptide. RT prediction of cross‐linked peptides using deep learning will require the design of new frameworks as well as new peptide encoding methods. Moreover, it may also be difficult to generate enough cross‐linked peptides for model training.

## 4 Deep Learning for MS/MS Spectrum Prediction

In a typical MS/MS‐based proteomics experiment, hundreds of thousands of MS/MS spectra can be generated. Information in an MS/MS spectrum generated from bottom‐up proteomics consists of mass‐to‐charge ratios (or *m*/*z*) and intensities of a set of fragment ions generated from digested peptides using methods like collision induced dissociation (CID), higher‐energy collisional dissociation (HCD) or electron‐transfer dissociation (ETD).^[^ ^52^ ^]^ The patterns of an MS/MS spectrum for a peptide (the *m*/*z* and intensities of fragment ions, and their types) are mainly determined by a few key factors including: 1) The type of MS instrument as well as the fragmentation method (e.g., CID, HCD, or ETD) used to fragment peptides and its setting, such as normalized collision energy (NCE), 2) peptide sequence, and 3) the precursor charge state of the peptide.^[^ ^29^, ^53^ ^]^ Peptide identification relies primarily on the patterns of these fragment ions. Although the mechanism underlying peptide fragmentation is complicated and still not well‐understood, these patterns are reproducible and, in general, predictable as demonstrated by many studies.^[^ ^54^, ^55^, ^56^, ^57^ ^]^

**Figure 2:**
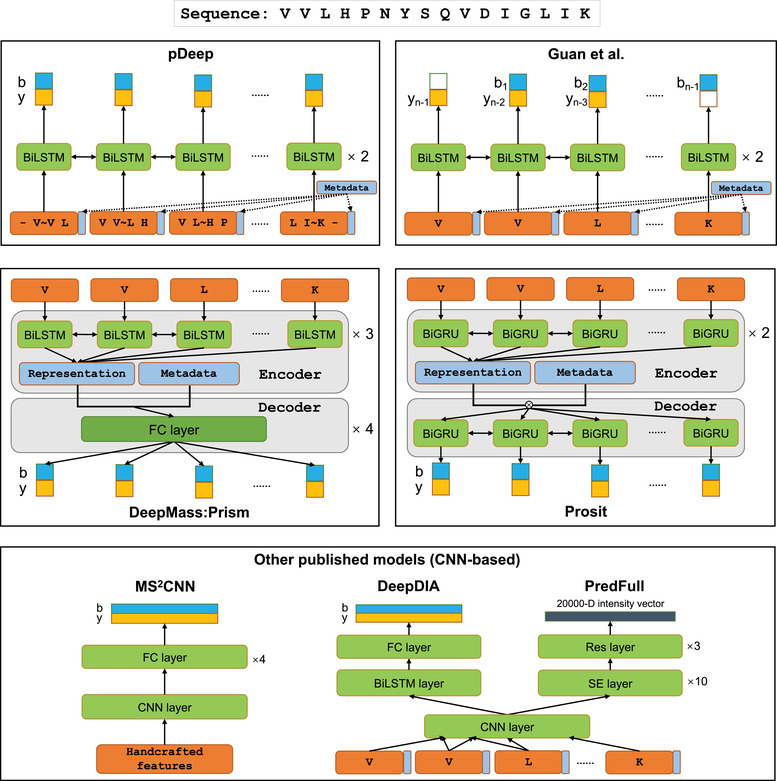
Brief network architectures of the deep learning tools for MS/MS spectrum prediction. FC layer refers to the fully connected layer, BiLSTM refers to bidirectional LSTM, and BiGRU refers to bidirectional GRU. For different models, metadata may include precursor charge state, precursor mass, collision energy, instrument type, etc. “∼” is the cleavage site.

A number of tools have been developed to predict MS/MS spectra from peptide sequences. These methods can be divided into hypothesis‐driven methods and data‐driven methods. Several hypothesis‐driven algorithms have been developed based on the mobile proton hypothesis, which is a widely accepted hypothesis to study peptide fragmentation pathways in tandem mass spectrometry.^[^ ^58^, ^59^, ^60^, ^61^ ^]^ MassAnalyzer is a popular tool in this category.^[^ ^58^ ^]^ Data‐driven methods, or more generally machine learning‐based methods, include traditional machine learning‐based tools, such as PeptideART,^[^ ^55^, ^62^ ^]^ MS^2^PIP,^[^ ^63^, ^64^, ^65^ ^]^ MS^2^PBPI,^[^ ^66^ ^]^ and other tools,^[^ ^67^, ^68^ ^]^ and deep learning‐based tools as shown in **Table** **2**, such as pDeep,^[^ ^57^, ^69^ ^]^ Prosit,^[^ ^29^ ^]^ DeepMass:Prism,^[^ ^45^ ^]^ MS^2^CNN,^[^ ^70^ ^]^ DeepDIA,^[^ ^30^ ^]^ Predfull^[^ ^56^ ^]^ and the model proposed in Guan et. al^[^ ^46^ ^]^ (**Figure** **2**). Deep learning models have been demonstrated to outperform both traditional machine learning models and hypothesis‐driven methods. The spectra predicted by deep learning models are highly similar to the experimental spectra. Remarkably, the similarities between deep learning predicted spectra and corresponding experimental spectra are very close to the average similarities between replicated experimental spectra for the same peptides.^[^ ^56^, ^57^ ^]^

**Table 2:** List of deep learning‐based MS/MS spectrum prediction tools. The fragment ion type supported by each tool is summarized based on its original publication and available trained models

No.	Software	Framework	Core network model	Fragment ion type	Usability^a)^	Year	Reference
1	pDeep/pDeep2	Keras/TensorFlow	RNN	b/y; c/z	O,C,P,T	2017/2019	[57, 69]
2	Prosit	Keras/TensorFlow	RNN	b/y	O,C,W,P,T	2019	[29]
3	DeepMass:Prism	Keras/TensorFlow	RNN	b/y	W	2019	[45]
4	Guan et al.	Keras/TensorFlow	RNN	b/y	O,C,P,T	2019	[46]
5	MS^2^CNN	Keras/TensorFlow	CNN	b/y	O,C,P	2019	[70]
6	DeepDIA	Keras/TensorFlow	CNN+RNN	b/y	O,C,P,T	2020	[30]
7	Predfull	TensorFlow	CNN	All possible ions at all *m*/*z* axises	O,C,W,P,T	2020	[56]

^a)^ O, open‐source; C, command line; P, provide trained model for prediction; W, web interface; T, provide option for model training. The link of each tool could be found at https://github.com/bzhanglab/deep_learning_in_proteomics.

pDeep consists of two BiLSTM layers followed by a time‐distributed fully connected output layer, and it takes a one‐hot encoded peptide sequence and corresponding precursor charge state of the peptide as inputs and outputs intensities of different fragment ion types at each position along the input peptide sequence^[^ ^57^ ^]^ (Figure 2). pDeep was first developed based on built‐in static LSTM APIs in Keras that only accept an input peptide sequence with a predefined fixed length (20 in the original paper). When a peptide sequence is shorter than the predefined length, “zeros” are padded into the sequence and masked by a masking layer. On the other hand, peptides that are longer than the predefined length will be discarded with no prediction. pDeep2 improves the original version by using the dynamic BiLSTM API in TensorFlow, which dynamically unrolls the recurrent cell based on the length of the input sequences to overcome the length limitation.^[^ ^69^ ^]^ Dynamic LSTM can also avoid the calculation for extra “zero” padded sequences, which potentially improves the prediction speed. In order to predict MS/MS spectrum for modified peptides without sufficient training data, pDeep2 uses transfer learning to train PTM models on top of the base model developed for unmodified peptides. The prediction performance for modified peptides is comparable to that for unmodified peptides. In pDeep2, a modification is represented as a feature vector of length eight based on its chemical composition (e.g., the chemical composition of phosphorylation which often occurs on serine (S), threonine (T), or tyrosine (Y) is HPO3, thus it is encoded as a feature vector [1,0,0,3,0,1,0,0]). This is similar to how modifications are encoded in DeepLC. With this encoding scheme, pDeep2 models can be used to predict spectra for peptides with modifications that are not present in the training data. However, the prediction performance for those peptides is very low without using transfer learning. In addition to one‐hot encoded peptide sequences, associated feature vectors of modifications, and corresponding precursor charge states of the peptides, other associated metadata including the instrument type and the collision energy are also encoded as inputs. Including peptide associated metadata in the modeling process allows the application of the resulted models to different MS instruments and settings, thus avoiding the need to train models for each combination of MS experiment parameters. The model used by Guan et al.^[^ ^46^ ^]^ for MS/MS spectrum prediction is similar to pDeep except for slightly different input and output structures.

Both Prosit and DeepMass:Prism are also BiRNN‐based networks. Prosit uses a BiGRU network, whereas DeepMass:Prism uses a BiLSTM network. Similar to pDeep2, peptide sequences along with associated metadata are encoded as input. A peptide sequence is encoded using one‐hot encoding in DeepMass:Prism, whereas it is represented as a discrete integer vector for feeding into an embedding layer of the network in Prosit. Both tools use a fixed length of peptide encoding. In other words, the trained models from the two tools cannot make predictions for any peptides with a length exceeding the longest peptide in the training data.

MS^2^CNN is based on CNN rather than RNN (LSTM or GRU). A single CNN model based on the network structure of LeNet‐5^[^ ^71^ ^]^ is constructed to predict MS/MS spectra for peptides of a specific length and precursor charge state. Unlike the above models, MS^2^CNN uses handcrafted features of peptides as input instead of learning peptide representation directly from peptide sequences. The features used in MS^2^CNN include peptide composition (similar to amino acid composition), mass‐to‐charge ratio (*m*/*z*), and peptide physicochemical properties such as isoelectric point, instability index, aromaticity, secondary structure fraction, helicity, hydrophobicity, and basicity. Because peptide associated metadata are not used in the modeling, the models can only be applied to data generated under matched experiment conditions.

DeepDIA uses a hybrid CNN and BiLSTM network for MS/MS spectra prediction. This model is similar to the one used for RT prediction in DeepDIA. A peptide sequence is encoded using one‐hot encoding. Separate models are required to be trained for different MS conditions and peptide precursor charge states.

All of the aforementioned methods aim to predict the intensities of expected backbone fragment ion types (e.g., b/y ions for CID and HCD spectra, c/z ions for ETD spectra, as well as their associated neutral losses). However, besides the backbone fragment ions, MS/MS spectra could contain many additional fragment ions that are derived from peptide fragmentation rather than background noise.^[^ ^56^, ^72^ ^]^ These fragment ions are typically ignored in spectra annotation and PSM scoring. A recent study showed that these fragment ions could account for ≈30% of total ion intensities in HCD spectra.^[^ ^56^ ^]^ Some of the ignored ions with high intensity may be informative and thus can be used to improve peptide identification. Predfull utilizes a generalized sequence‐to‐sequence model based on the structure of the residual CNN and a multitask learning strategy to predict the intensities for all possible *m*/*z* from peptide sequences without assumptions or expectations on which kind of ions to predict.^[^ ^56^ ^]^ Each MS/MS spectrum in the training data is represented as a sparse 1D vector by binning the *m*/*z* range between 180 and 2000 with a given bin width so that all the peaks in an MS/MS spectrum are used in the training. This is fundamentally different from other tools in which only the annotated backbone ions are used for training. In addition, a multitask learning strategy is used in Predfull to improve the prediction accuracy for spectra with insufficient training data (e.g., 1+ and 4+ HCD spectra and ETD spectra of all charges). Predfull showed better performance than the backbone‐only spectrum predictors (pDeep, Prosit and DeepMass:Prism).

Accurately predicted MS/MS spectra from peptide sequences have promising applications. First, they can be used to improve protein identification in DDA data analysis. For database searching, the predicted MS/MS spectra can be used either in the scoring of PSMs by a search engine^[^ ^45^ ^]^ or in PSM rescoring using post‐processing tools such as Percolator.^[^ ^29^, ^51^, ^73^ ^]^ For spectral library searching, accurately predicted MS/MS spectra can lead to comprehensive high quality spectra libraries. For de novo peptide sequencing, deep learning‐based MS/MS spectrum prediction could be useful in ranking candidate peptides.^[^ ^74^ ^]^

Next, predicted MS/MS spectra combined with RT prediction can be used to build a spectral library in silico in DIA data analysis or the method development in targeted proteomics experiments (e.g., MRM or PRM experiments). A spectral library mainly contains the peptide RT and peptide fragment ions and their intensities, and both can be predicted accurately using deep learning methods. Traditionally, such a spectral library is built based on peptide identifications from conventional DDA experiments, which often involve offline pre‐fractionation of peptide samples to improve the coverage of the library.^[^ ^75^, ^76^, ^77^ ^]^ Therefore, it requires extra instrument time and cost to generate such a library for DIA data analysis because of the complex mixtures of peptides in DIA MS2 scans. Moreover, such a library still suffers from the limitations of DDA experiments for peptide identification. In addition, generating such a library for peptides with PTMs such as phosphorylation is challenging. Recently, a few studies have demonstrated the potential of deep learning‐based MS/MS spectrum prediction in DIA library generation.^[^ ^29^, ^30^, ^31^, ^32^, ^78^ ^]^ We expect in silico spectral library generation using deep learning will become increasingly popular in DIA data analysis. In targeted proteomics experiments, the predicted spectral library is especially useful to guide the method development (e.g., transition list design in MRM assays) for detecting proteins with low abundance or novel proteins that are typically difficult to detect in DDA experiments.

Finally, deep learning‐based MS/MS spectrum prediction can enhance our understanding of the principles behind peptide fragmentation. For example, Tiwary et al.^[^ ^45^ ^]^ reported that the outputs from DeepMass:Prism can indicate the fragmentation efficiencies between different amino acid pairs. In addition, in order to study the influence between each amino acid in the peptide sequence and the predicted intensity of each peak, the method of integrated gradients^[^ ^79^ ^]^ was used to attribute predictions from DeepMass:Prism to specific input amino acids. Zhou et al.^[^ ^57^ ^]^ showed that accurate prediction of a spectrum by deep learning enables discrimination of isobaric amino acids, such as I versus L, GG versus N, AG versus Q, KR versus RK, etc. Furthermore, Guan et al.^[^ ^46^ ^]^ showed that the discriminative power for isomeric peptides is higher when isobaric amino acid‐related local ion similarities are considered.

Each tool has its own strengths and weaknesses. Comprehensive independent benchmarking of existing tools is essential to guide the selection of methods for real applications. Recently, Xu et al.^[^ ^80^ ^]^ benchmarked three deep learning‐based tools (Prosit, pDeep2 and Guan's work^46^) and one traditional machine learning‐based tool (MS^2^PIP) for MS/MS spectra prediction. The results showed that the deep learning‐based tools outperform MS^2^PIP and the performance of deep learning‐based tools may vary across different datasets and different peptide precursor charge states.

Although significant improvements have been made for MS/MS spectrum prediction using deep learning from peptide sequences, there is still much room for improvement in the prediction for peptides with modifications. Most of the current MS/MS spectrum prediction models are mainly developed for unmodified peptides, and most of the existing models cannot be directly used to predict peptides with modifications not present in the training data. Although some of the current tools can be trained with peptides containing modifications of interest, specific training strategies such as transfer learning are required to achieve satisfactory prediction performance because of the small size of available training data with specific modifications. This situation is similar to RT prediction for peptides with modifications. In pDeep2, Zeng et al.^[^ ^69^ ^]^ have demonstrated that the transfer learning strategy could significantly improve the prediction for modified peptides with limited training examples. However, transfer learning is only well supported in pDeep2. Furthermore, for some PTMs like glycosylation, the prediction of MS/MS spectra for intact glycopeptides would be more challenging due to the complexity of intact glycopeptides and the lack of large experimental high quality MS/MS spectra from intact glycopeptides. In addition, all current deep learning‐based tools are developed for MS/MS spectrum prediction of single peptides in which a predicted spectrum corresponds to a single linear peptide. As with RT prediction, MS/MS spectra prediction for cross‐linked peptides will require new frameworks and new peptide encoding methods.

## 5 Deep Learning for De Novo Peptide Sequencing

**Figure 3:**
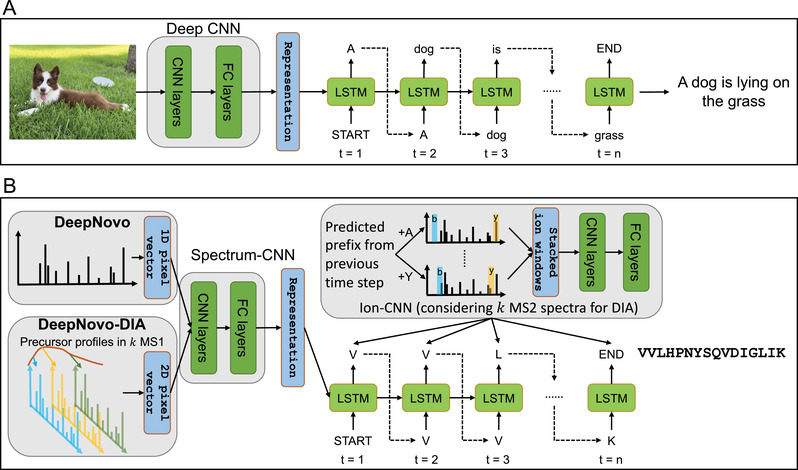
From image captioning to DeepNovo. A) A typical neural network architecture of image captioning. B) The neural network architectures of DeepNovo and DeepNovo‐DIA.

Another breakthrough application of deep learning in the field of proteomics is de novo peptide sequencing, as demonstrated in DeepNovo.^[^ ^10^ ^]^ In de novo peptide sequencing, the peptide sequence is directly inferred from an MS/MS spectrum without relying on a protein database. If we regard an MS/MS spectrum as an image and the peptide sequence as an image description, de novo peptide sequencing bears some similarity to deep learning‐based image captioning,^[^ ^81^, ^82^ ^]^ which is the task of generating a description in a specific language for a given image. Encoder‐decoder architecture is one of the widely used architectures in deep learning‐based image captioning, where an image‐CNN layer is typically used to encode the image into a hidden representation, and an RNN (e.g., LSTM) layer is used to decode and predict the words one by one to form sentences of a language (**Figure** **3**A). DeepNovo views the input spectrum as an image and the output peptide sequence as a sentence of a protein language.

More specifically, DeepNovo first discretizes a spectrum into an intensity vector with length 500 000 (high‐resolution data, 0.01 Da per pixel/bin, up to 5000 Da) or 50 000 (low‐resolution data, 0.1 Da per pixel/bin, up to 5000 Da). It then uses a spectrum‐CNN as an encoder for the intensity vector, and an LSTM as a decoder. In order to capture amino acid signals in the spectrum, the intensity vector is further processed using an amino acid‐mass shift operation before being fed into the spectrum‐CNN. The outputs of the spectrum‐CNN are then passed into the decoder. The LSTM model aims to predict the probabilities of all considered amino acids at each position of a peptide sequence. Specifically, at position *t*, LSTM takes the previously predicted amino acid and previous hidden state at position *t* − 1 as input to predict the probabilities of the next amino acids. In addition, an ion‐CNN model is used to learn features of fragment ions in a spectrum, and the outputs are combined with the LSTM model and run step by step starting from an empty sequence and ending with the full peptide sequence. Because the model has no way to restrict the mass of the predicted peptide sequence within the tolerance window of the precursor mass, DeepNovo uses the knapsack dynamic programming algorithm to quickly filter out any “precursor‐unreachable” amino acid prediction at position *t*, and only considers “precursor‐reachable” predictions (informally, a prediction at any position is “precursor‐unreachable”, if the predicted subsequence can never reach the precursor mass by considering any amino acid combinations, otherwise it is “precursor‐reachable”). After combining the spectrum‐CNN, LSTM, ion‐CNN, and knapsack, DeepNovo outperforms PEAKS,^[^ ^83^ ^]^ Novor,^[^ ^84^ ^]^ and PepNovo^[^ ^85^ ^]^ in terms of recall on both the peptide and amino acid levels, showing the extraordinary ability of DeepNovo.

DeepNovo was later extended to DeepNovo‐DIA to perform de novo sequencing on DIA data.^[^ ^86^ ^]^ The basic framework of DeepNovo‐DIA is similar to DeepNovo. In DIA data, there are multiple MS/MS spectra associated with a given precursor ion, and each MS/MS spectrum typically contains fragment ions from multiple peptides. DeepNovo‐DIA stacks these spectra along the retention time dimension to form a 2D intensity vector. For a given precursor ion, besides its associated MS/MS spectra, its MS1 intensity profile is also encoded to feed into the network. The correlation between the precursor and its fragment ions could be learned in the ion‐CNN module of DeepNovo‐DIA. For the traditional de novo sequencing algorithm, it is not an easy task to redesign the algorithm to support DIA data analysis due to the high complexity of DIA spectra. In contrast, data‐driven DeepNovo can perform DIA data analysis after only redesigning the partial architecture to utilize the extra dimensionality of DIA data (*m*/*z* and retention time). However, validating the results of de novo sequencing from DIA data is quite difficult and is still an open problem.

Recently, Karunratanakul et al.^[^ ^87^ ^]^ developed SMSNet to further improve de novo peptide sequencing using deep learning. The deep learning architecture of SMSNet is similar to that used in DeepNovo. A key innovation in SMSNet is the use of the multi‐step Sequence‐Mask‐Search strategy. For traditional de novo sequencing algorithms, Muth et.al. showed that most of incorrect peptide predictions are from locally incorrect short subsequences.^[^ ^88^ ^]^ Local incorrectness is also a problem in deep learning‐based de novo sequencing. The multi‐step Sequence‐Mask‐Search strategy addresses this problem. More specifically, SMSNet further uses a rescoring network after the encoder‐decoder network to estimate the confidence score of each amino acid of the predicted sequence. If unconfident local amino acids are detected, SMSNet corrects them by querying a protein sequence database, resulting in higher sequencing accuracies. It has been shown that this strategy could effectively improve the accuracy of SMSNet. Since it relies on the protein sequence database, the performance of SMSNet may be limited by the quality and completeness of the database provided. SMSNet outperforms DeepNovo on a few different datasets and has shown promising application in immunopeptidomics.^[^ ^87^ ^]^

The purely data‐driven deep learning model is not the only way to improve the performance of de novo peptide sequencing. By considering the predicted spectra based on deep learning, pNovo3 re‐ranks the peptide candidates generated by pNovo+ (a spectrum‐graph and dynamic programming based algorithm^[^ ^89^ ^]^) using a learning‐to‐rank framework, leading to higher accuracies than DeepNovo.^[^ ^74^ ^]^ This example shows that coupled with deep learning, traditional de novo sequencing can be improved as well. In conclusion, deep learning has opened a new perspective for de novo peptide sequencing.

Clear improvement has been achieved using deep learning compared with previous de novo peptide sequencing methods. This has led to a few promising applications using de novo peptide sequencing including complete de novo sequencing of antibody sequences and discovery of new the human leukocyte antigen (HLA) antigens.^[^ ^10^, ^87^, ^90^ ^]^ However, there is still a huge gap between de novo peptide sequencing and database search based peptide identification methods in terms of accuracy of peptide identification, thus resulting in its limited applications in proteomics studies. Further improvement could be achieved from, but not limited to, the following aspects. First, training using larger datasets may further improve the models.^[^ ^91^ ^]^ The second aspect is training species‐specific models using transfer learning by leveraging large datasets from other species. Since the protein sequences from different species may have different patterns, the deep learning models trained using MS/MS data from one species may not generalize well to another species, but the patterns and rules learned from other species with large datasets could benefit the training for a specific species with a relatively small dataset. The third aspect is extensively optimizing the current deep learning architectures using hyperparameter tuning methods or designing more efficient architectures using neural architecture search algorithms.

## 6 Deep Learning for Post‐Translational Modification Prediction

Over 300 types of PTMs are known to occur physiologically across different proteins.^[^ ^92^ ^]^ PTMs tremendously increase the complexity of cellular proteomes, diversify protein functions, and play important roles in many biological processes.^[^ ^93^, ^94^ ^]^ PTMs can be experimentally identified in both low‐throughput experiments and high‐throughput MS‐based experiments.^[^ ^95^ ^]^ In addition, computational algorithms can also be used to predict PTM sites. Machine learning is the primary approach used for PTM prediction because of its flexibility and performance. The prediction of a specific type of PTM site, such as phosphorylation, can be formulated as two classification tasks. The first, referred to as general site prediction, is to predict whether a given site can be modified, such as by being phosphorylated. The second is to predict whether a given site can be modified by a specific enzyme, such as a specific kinase for phosphorylation, referred to as enzyme‐specific prediction.

Deep learning has been used in the prediction of PTM sites for phosphorylation,^[^ ^96^, ^97^, ^98^, ^99^, ^100^ ^]^ ubiquitination,^[^ ^101^, ^102^ ^]^ acetylation,^[^ ^103^, ^104^, ^105^, ^106^ ^]^ glycosylation,^[^ ^107^ ^]^ malonylation,^[^ ^108^, ^109^ ^]^ succinylation,^[^ ^110^, ^111^ ^]^ glycation,^[^ ^112^ ^]^ nitration/nitrosylation,^[^ ^113^ ^]^ crotonylation^[^ ^114^ ^]^ and other modifications^[^ ^115^, ^116^, ^117^, ^223^ ^]^ as shown in **Table** **3**. MusiteDeep, the first deep learning‐based PTM prediction tool, provides both general phosphosite prediction and kinase‐specific phosphosite prediction for five kinase families, each with more than 100 known substrates.^[^ ^96^ ^]^ MusiteDeep uses one‐hot encoding of a 33‐amino acid sequence centered at the prediction site (i.e., 16 amino acids flanking on each side of the site) as input where phosphorylation sites on S, T, or Y annotated by UniProt/Swiss‐Prot are used as positive data, whereas the same amino acid excluding annotated phosphorylation sites from the same proteins are regarded as negative data. The input data are fed into a multi‐layer CNN for classification. For kinase‐specific site prediction, transfer learning from the base general phosphosite model is used to train models for each kinase. The kinase‐specific models make use of the general feature representations learned from the model developed on general phosphorylation data. This approach could also reduce possible overfitting caused by the limited numbers of kinase‐specific substrates. In the original study, MusiteDeep was shown to outperform a few non‐deep learning‐based tools for general site prediction, including Musite,^[^ ^118^ ^]^ NetPhos 3.1,^[^ ^119^ ^]^ ModPred,^[^ ^120^ ^]^ and PhosPred‐RF.^[^ ^121^ ^]^ It also outperformed Musite, NetPhos 3.1, GPS 2.0,^[^ ^122^ ^]^ and GPS 3.0^[^ ^123^ ^]^ for kinase‐specific prediction in most cases. Another phosphosite prediction tool, DeepPhos, uses densely connected CNN blocks including both intra block concatenation layers and inter block concatenation layers to make final phosphorylation predictions.^[^ ^97^ ^]^ This network architecture aims to capture multiple representations of sequences. Similar to MusiteDeep, DeepPhos also utilizes the transfer learning strategy to perform kinase‐specific prediction. DeepPhos was shown to outperform MusiteDeep in both general site and kinase‐specific predictions. In contrast to MusiteDeep and DeepPhos in which a model is trained for each kinase independently, in a more recent tool, EMBER, a single unified multi‐label classification model was trained to predict phosphosites for multiple kinase families using deep learning.^[^ ^99^ ^]^ In EMBER, a 15‐amino acid sequence centered at the prediction site is first encoded as input not only using one‐hot encoding but also using embedding based on a Siamese neural network.^[^ ^124^ ^]^ The Siamese network, which comprises of two identical LSTM networks with identical hyperparameters as well as learned weights, is used to learn a semantically meaningful vector representation for each sequence. Each LSTM network takes a different peptide as input and the two networks are joined at the final layer. The two types of encoded sequences are then fed into respective CNNs, which have identical hyperparameters. The CNNs are concatenated in the final layer, followed by a series of fully connected layers. The output is an eight‐element vector, in which each value corresponds to the probability of the input site being phosphorylated by a kinase family. In order to leverage evolutionary relationships between kinase families in the modeling, a kinase phylogenetic metric is calculated and used via a kinase phylogeny‐based loss function.

**Table 3:** List of deep learning‐based PTM prediction tools

No.	Software	PTM	Framework	Core network model	Group^b)^	Window size	Input encoding	Usability^c)^	Year	Reference
1	DeepAce	Acetylation	Keras/Theano	CNN+DNN	G	53/29	One‐hot, handcrafted features	O,C,T	2018	[103]
2	Deep‐PLA	Acetylation	Keras/TensorFlow	DNN	E	31	Handcrafted features	W,P	2019	[104]
3	DeepAcet	Acetylation	TensorFlow	DNN	G	31	One‐hot, BLOSUM, handcrafted features	O	2019	[106]
4	DNNAce	Acetylation	Keras/TensorFlow	DNN	G	13/17/21	One‐hot, BLOSUM, handcrafted features	O	2020	[105]
5	pKcr	Crotonylation	Keras/TensorFlow	CNN	G	29	Word embedding	W,P	2020	[114]
6	DeepGly	Glycation	‐^a)^	CNN+RNN	G	49	Word embedding	‐	2019	[112]
7	Long et al.	Hydroxylation	MXNet	CNN+RNN	G	13	Handcrafted features	‐	2018	[115]
8	MUscADEL	Lysine PTMs	‐	RNN	G	27/1000	Word embedding	W,P	2018	[224]
9	LEMP	Malonylation	TensorFlow	RNN+RF	G	31	Word embedding, handcrafted features	W,P	2019	[108]
10	DeepNitro	Nitration/Nitrosylation	Deeplearning4j	DNN	G	41	handcrafted features	C,W,P	2018	[113]
11	MusiteDeep	Multiple	Keras/TensorFlow	CNN	E, G	33	One‐hot	O,C,W,P,T	2017/2020	[96, 128]
12	NetPhosPan	Phosphorylation	Lasagne/Theano	CNN	E	21	BLOSUM	C,W,P	2018	[98]
13	DeepPhos	Phosphorylation	Keras/TensorFlow	CNN	E, G	15/33/51	One‐hot	O,C,P,T	2019	[97]
14	EMBER	Phosphorylation	PyTorch	CNN+RNN	E	15	One‐hot	O,C,T	2020	[99]
15	DeepKinZero	Phosphorylation	TensorFlow	ZSL	E	15	Word embedding	O,C,P,T	2020	[100]
16	CapsNet_PTM	Multiple	Keras/TensorFlow	CapsNet	G	33	Handcrafted features	O,C,T	2018	[117]
17	GPS‐Palm	S‐palmitoylation	Keras/TensorFlow	CNN	G	21	Handcrafted features	G,P	2020	[116]
18	CNN‐SuccSite	Succinylation	‐	CNN	G	31	Handcrafted features	W,P	2019	[111]
19	DeepUbiquitylation	Ubiquitination	Keras/Theano	CNN+DNN	G	49	One‐hot, handcrafted features	O,C,P,T	2018	[102]
20	DeepUbi	Ubiquitination	TensorFlow	CNN	G	31	One‐hot, handcrafted features	O	2019	[101]

^a)^ Framework used in the tool is not available in the original study;^b)^ G, general site prediction; E, enzyme‐specific site prediction;

^c)^ O, open‐source; G, graphical user interface; C, command line; P, provide trained model for prediction; W, web interface; T, provide option for model training. The link of each tool could be found at https://github.com/bzhanglab/deep_learning_in_proteomics.

A common limitation of MusiteDeep, DeepPhos, and EMBER is that they only predict phosphorylation sites for a limited number of kinases with sufficient numbers of known substrate phosphosites. However, among over 500 protein kinases described in the human proteome, only a small fraction have more than 30 annotated substrate phosphosites,^[^ ^98^, ^100^, ^125^ ^]^ and more than 95% of the known phosphosites have no known upstream kinases.^[^ ^125^, ^126^ ^]^ In order to address this limitation, a few deep learning methods have been developed to enable the prediction of phosphorylation sites for kinases characterized by limited or no experimental data.^[^ ^98^, ^100^ ^]^ Inspired by the pan‐specific method for MHC‐peptide binding prediction (see next Section), Fenoy et al.^[^ ^98^ ^]^ proposed a CNN framework, NetPhosPan, to develop a pan‐kinase‐specific prediction model to enable kinase‐specific predictions for any kinase with a known protein sequence. In addition to requiring peptide sequences with the amino acids S, T, or Y in the center as input, NetPhosPan also requires kinase domain sequences to train a single model for kinase‐specific predictions. Both peptide sequences and kinase domain sequences are encoded using the BLOSUM matrix. In this way, the model can leverage information between different kinases to enable the predictions for kinases without known sites and improve the predictions for kinases with few known sites.

DeepKinZero^[^ ^100^ ^]^ is the first zero‐shot learning (ZSL) tool to predict the kinase which can phosphorylate a given site for kinases without known substrates or unseen kinases in training. Zero‐shot learning is a type of machine learning method that can deal with recognition tasks for classes without training examples.^[^ ^127^ ^]^ The key idea underlying DeepKinZero is to recognize a target site of a kinase without any known site through transferring knowledge from kinases with many known sites to this kinase by establishing a relationship between the kinases using relevant auxiliary information such as functional and sequence characteristics of the kinases. Similar to NetPhosPan, both substrate sequences and kinase sequences are required as input, and they are encoded and fed into the zero‐shot learning framework for training. The kinases are encoded using a few different methods including kinase taxonomy and distributed representation of their kinase domain sequences using ProtVec,^[^ ^12^ ^]^ which is different from the BLOSUM matrix encoding used in NetPhosPan.

Although phosphorylation is the most widely studied PTM, deep learning has also been applied to other PTMs as shown in Table 3. Most of the tools for the other PTMs are general PTM site prediction tools rather than enzyme‐specific tools. A major advantage of deep learning is to learn representation efficiently from peptide sequences without handcrafted features for PTM site prediction. However, handcrafted features can also be fed into deep neural networks just as for traditional machine learning algorithms for classification. For example, He et al.^[^ ^102^ ^]^ proposed a multimodal deep architecture in which one‐hot encoding of peptide sequences as well as physicochemical properties and sequence profiles were fed into a deep neural network for lysine ubiquitination prediction. The authors showed that the multimodal model outperformed the model using one‐hot encoding of peptide sequences alone as input. Additionally, Chen et al.^[^ ^108^ ^]^ found that combining a word‐embedded LSTM‐based classifier with a traditional RF model encoding the amino acid frequency improved the prediction of malonylation sites. Most of the tools shown in Table 3 are developed for the prediction of one type of PTM site. A few tools are developed for the prediction of multiple types of PTM sites. One is CapsNet_PTM which uses a CapsNet for seven types of PTMs prediction.^[^ ^117^ ^]^ Most recently, MusiteDeep has been extended to incorporate the CapsNet with ensemble techniques for the prediction of more types of PTM sites.^[^ ^128^ ^]^ The tool could be easily extended to predict more PTMs given enough number of known sites for training.

Advances in MS‐based PTM profiling have enabled the identification and quantification of PTMs at the proteome scale,^[^ ^95^, ^129^ ^]^ and PTM profiling datasets are growing rapidly.^[^ ^130^ ^]^ The large number of sites identified in these studies will eventually lead to accurate general site prediction models for many PTM types. The accuracy of these models relies on high‐quality site identifications in these high‐throughput experiments, an area for future development. Moreover, MS‐based profiling cannot provide direct evidence for enzyme‐substrate relationships for PTMs. It remains a big challenge to experimentally generate a large number of high‐quality enzyme‐substrate relationships for different types of PTMs to facilitate the training and evaluation of enzyme‐specific prediction models.

## 7 Deep Learning for MHC‐Binding Peptide Prediction

MHC (called human leukocyte antigen, or HLA, in humans) class I and class II genes encode cell surface proteins that present self and foreign peptides for inspection by T cells and thus play a critical role in generating immune responses.^[^ ^131^ ^]^ Peptides derived from intracellular proteins are predominantly presented by MHC class I molecules, whereas peptides presented by MHC class II molecules are usually of extracellular origin.^[^ ^132^ ^]^ MHC genes are highly polymorphic, and it is important to know which peptides can be presented by a specific MHC allele. There are two types of experimental assays for identifying MHC‐binding peptides, in vitro peptide binding assays and MS/MS analysis of MHC‐bound peptides (immunopeptidomics).^[^ ^133^ ^]^ The primary database for in vitro binding assay data is the Immune Epitope Database (IEDB),^[^ ^134^ ^]^ and immunopeptidomics data can be found in IEDB, SysteMHC Atlas,^[^ ^135^, ^136^ ^]^ or new publications describing large multi‐allelic or single‐allelic datasets.^[^ ^137^, ^138^, ^139^ ^]^ Based on these data, many computational methods have been developed to predict MHC‐binding peptides.^[^ ^140^ ^]^

Computational methods for MHC‐peptide binding prediction can be grouped into allele‐specific and pan‐specific methods. Because biological samples used for immunopeptidomics analysis typically carry multiple MHC alleles, allele‐specific models are typically trained with in vitro peptide binding assay data, and one prediction model is constructed for each MHC allele separately. Allele‐specific models usually perform well for common MHC alleles for which a large amount of experimental data is available for model training; however, models for alleles with limited experimental data are less reliable. To address this data scarcity issue, pan‐specific methods have been proposed. Typically, a single pan‐specific model is trained using data from all alleles, and the trained model can be applied to alleles with few training samples and even alleles not included in the training data. Allele‐specific models are more accurate when restricted to certain alleles with a large number of training samples, while pan‐specific models deliver more stable and better overall performances when applied to MHC alleles with limited or no in vitro peptide binding assay data.^[^ ^141^ ^]^

During the past few years, a number of deep learning‐based methods have been developed that outperform traditional machine learning methods, including shallow neural networks, for peptide‐MHC binding prediction (**Table** **4**). Among these algorithms, 14 (ConvMHC,^[^ ^142^ ^]^ HLA‐CNN,^[^ ^143^ ^]^ DeepMHC,^[^ ^144^ ^]^ DeepSeqPan,^[^ ^145^ ^]^ MHCSeqNet,^[^ ^146^ ^]^ MHCflurry,^[^ ^147^ ^]^ DeepHLApan,^[^ ^148^ ^]^ ACME,^[^ ^149^ ^]^ EDGE,^[^ ^137^ ^]^ CNN‐NF,^[^ ^150^ ^]^ DeepNeo,^[^ ^151^ ^]^ DeepLigand,^[^ ^152^ ^]^ MHCherryPan,^[^ ^153^ ^]^ and DeepAttentionPan^[^ ^141^ ^]^) are specific for MHC class I binding prediction, three (DeepSeqPanII,^[^ ^154^ ^]^ MARIA,^[^ ^138^ ^]^ and NeonMHC2^[^ ^139^ ^]^) are specific for MHC class II binding prediction, and four (AI‐MHC,^[^ ^155^ ^]^ MHCnuggets,^[^ ^156^ ^]^ PUFFIN,^[^ ^157^ ^]^ and USMPep^[^ ^158^ ^]^) can make predictions for both classes. All four types of peptide encoding approaches illustrated in Figure 1 are used in these tools, with one‐hot encoding and BLOSUM matrix encoding being the most frequently used methods (Table 4).

**Table 4:** List of deep learning‐based MHC‐peptide binding prediction tools

No.	Software	MHC Type	Core network model	Framework	Group	Data Type^a)^	Input encoding	Usability^b)^	Year	Reference
1	ConvMHC	MHC Class I	CNN	Keras	Pan‐specific	BA	Handcrafted features	W,P	2017	[142]
2	HLA‐CNN	MHC Class I	CNN	keras/Theano	Allele‐specific	BA	Word embedding	O,C,T	2017	[143]
3	DeepMHC	MHC Class I	CNN	‐	Allele‐specific	BA	One‐hot	‐	2017	[144]
4	DeepSeqPan	MHC Class I	CNN	Keras/Tensorflow	Pan‐specific	BA	One‐hot	O,C,P,T	2019	[145]
5	AI‐MHC	MHC Class I/II	CNN	TensorFlow	Pan‐specific	BA	Word embedding	W,P	2018	[155]
6	DeepSeqPanII	MHC Class II	CNN+RNN	PyTorch	Pan‐specific	BA	One‐hot + BLOSUM	O,C,P,T	2019	[154]
7	MHCSeqNet	MHC Class I	RNN	Keras/Tensorflow	Pan‐specific	BA	Word embedding	O,C,P,T	2019	[146]
8	MARIA	MHC Class II	RNN	Keras/Tensorflow	Pan‐specific	BA + MS	One‐hot	W,P	2019	[138]
9	MHCflurry	MHC Class I	CNN	Keras/Tensorflow	Allele‐specific	BA + MS	BLOSUM	O,C,P,T	2018	[147]
10	DeepHLApan	MHC Class I	RNN	Keras/Tensorflow	Pan‐specific	BA + MS	Word embedding	O,C,W,P	2019	[148]
11	ACME	MHC Class I	CNN	Keras/Tensorflow	Pan‐specific	BA	BLOSUM	O,C,P,T	2019	[149]
12	EDGE	MHC Class I	DNN	Keras/Theano	Allele‐specific	MS	One‐hot	O	2019	[137]
13	CNN‐NF	MHC Class I	CNN	MXNet	Allele‐specific	BA + MS	Handcrafted features	O	2019	[150]
14	MHCnuggets	MHC Class I/II	RNN	Keras/Tensorflow	Allele‐specific	BA + MS	One‐hot	O,C,P,T	2019	[156]
15	DeepNeo	MHC Class I	CNN	Theano	Pan‐specific	BA	2D interaction map	‐	2020	[151]
16	DeepLigand	MHC Class I	CNN	PyTorch	Pan‐specific	BA + MS	Word embedding + BLOSUM + One‐hot	O,C,P,T	2019	[152]
17	PUFFIN	MHC Class I/II	CNN	PyTorch	Pan‐specific	BA	One‐hot + BLOSUM	O,C,P,T	2019	[157]
18	NeonMHC2	MHC Class II	CNN	Keras/Tensorflow	Allele‐specific	MS	Handcrafted features	O,C,W,P,T	2019	[139]
19	USMPep	MHC Class I/II	RNN	PyTorch	Allele‐specific	BA	Word embedding	O,T	2020	[158]
20	MHCherryPan	MHC Class I	CNN+RNN	Keras/Tensorflow	Pan‐specific	BA	BLOSUM	‐	2019	[153]
21	DeepAttentionPan	MHC Class I	CNN	PyTorch	Pan‐specific	BA	BLOSUM	O,C,P,T	2019	[141]

^a)^ BA, binding assay data; MS, eluted ligand data from mass spectrometry experiments;

^b)^ O, open‐source; C, command line; P, provide trained model for prediction; W, web interface; T, provide option for model training. The link of each tool could be found at https://github.com/bzhanglab/deep_learning_in_proteomics.

In terms of the neural network architecture, 13 out of the 21 tools use CNN, five use RNN, two use both CNN and RNN, and one uses DNN. Remarkably, 13 out of the 21 make pan‐specific predictions, in which both the peptide and MHC protein sequence (either the pseudo sequence or the full sequence) are fed into the neural networks simultaneously for modeling. The interaction specificity between the peptide and the MHC molecule is thus learned during the training process.

As shown in Table 4, most of the effort in the field has focused on using in vitro binding assay data to predict binding affinity between an MHC molecule and a given peptide sequence. There are multiple upstream biological processes involved in the generation of these peptides. For example, cytosolic proteins need to be degraded by the 26S proteasome to create peptide fragments of an appropriate size. Only a subset of these peptides can be transported into the endoplasmic reticulum through transporter associated with antigen processing proteins, where they may be further trimmed by the aminopeptidases ERAP1 and ERAP2 before loading onto MHC class I molecules.^[^ ^159^ ^]^ Therefore, even if a peptide has strong MHC binding affinity in an in vitro binding assay, it may be not presentable without appropriate upstream configurations. Immunopeptidomics addresses this limitation by analyzing the naturally presented MHC binding peptides (also called eluted ligands). Here we focus on the tools that leverage immunopeptidomics data for predictive modeling.

Six of these tools use immunopeptidomics data in combination with binding assay data. In MHCflurry,^[^ ^147^ ^]^ MHC‐I immunopeptidomics data are used in either model selection (MHCflurry 1.2) or as training data in combination with binding assay data (MHCflurry train‐MS). In MARIA,^[^ ^138^ ^]^ binding affinity data are used to train a pan‐specific RNN model to generate peptide‐MHC binding affinity scores, and immunopeptidomics data are used to train a DNN model to estimate peptide cleavage scores. The full MARIA model is then trained using immunopeptidomics data combined with gene expression data as well as the two types of scores for predicting the likelihood of antigen presentation in the context of specific MHC‐II alleles. In CNN‐NF,^[^ ^150^ ^]^ binding affinity data are converted to binary data first and then combined with immunopeptidomics data for training using a CNN network. In DeepHLApan,^148^ both types of data are used in a similar way to CNN‐NF. In MHCnuggets,^[^ ^156^ ^]^ an LSTM model is first trained using binding affinity data for each MHC allele. A new network initiated with weights transferred from the first step is further trained with immunopeptidomics data when available. In DeepLigand,^152^ two modules are combined to predict MHC‐I peptide presentation rather than binding affinity prediction. The first module is a pan‐specific binding affinity prediction module based on a deep residual network while the second one is a peptide embedding module based on a deep language model (ELMo^[^ ^160^ ^]^). The peptide embedding module is trained using immunopeptidomics data separately to capture the features of eluted ligands. The outputs from the two modules are concatenated and then fed into a fully connected layer. Finally the affinity module and the fully connected layer are jointly trained using both binding affinity data and immunopeptidomics data to predict MHC‐I peptide presentation.

The other two tools use immunopeptidomics data alone. EDGE^[^ ^137^ ^]^ is trained on eluted ligand data bound to HLA class I molecules from MS experiments on multi‐allelic cancer samples. The algorithm also incorporates other information including gene expression levels, proteasome cleavage preferences (flanking sequences), protein and sample information. EDGE does not require explicit eluted ligand‐MHC allele paired data. More specifically, a peptide associated with multiple MHC alleles from a given biological sample is taken as a sample during the training. For the tool NeonMHC2,^[^ ^139^ ^]^ allele‐specific models based on CNN are trained using eluted ligand data from individual MHC alleles for more than 40 HLA‐II alleles. The authors specifically generated mono‐allelic data for model training and showed that the models trained on the mono‐allelic data are superior to allele‐specific binding predictors on deconvoluted multi‐allelic MS data and NetMHCIIpan.^[^ ^139^ ^]^

Superior performance has been shown for each of the published MHC‐binding peptide prediction tools in the original studies. However, because each of these tools has its own strengths and weaknesses, a systematic evaluation of these tools is urgently needed to guide method selection for real applications. As it has been demonstrated that incorporating immunopeptidomics data could significantly improve the performance of MHC peptide binding prediction, we expect that model performance will be further improved with rapidly growing immunopeptidomics data. For example, applying deep learning to a recently published large MHC‐I peptidome dataset from 95 HLA‐A, ‐B, ‐C, and ‐G mono‐allelic cell lines^[^ ^161^ ^]^ may enable accurate allele‐specific predictions for many MHC alleles. However, most of the public immunopeptidomics data are from samples with multiple MHC alleles. How to make full use of this type of data in the training of allele‐specific MHC peptide binding prediction models is an interesting, yet not well studied question. Besides increasing the size of training examples for individual MHC alleles, it has been shown that both source gene expression and cleavage preference information of antigen peptides are useful in MHC peptide binding prediction.^[^ ^137^, ^138^, ^161^ ^]^ We expect these information will be utilized in more tools in the future.

## 8 Deep Learning for Protein Structure Prediction

Protein structures largely determine their functions. Predicting spatial structure from amino acid sequence has significant applications in protein design and drug screening, among others.^[^ ^162^ ^]^ Structural genomics/proteomics projects were initiated to experimentally solve 3D structures of proteins on a large scale, and aimed to increase the coverage of structure space by targeting unrepresented families.^[^ ^163^ ^]^ Although over 13 500 structures have been deposited in Protein Data Bank (PDB) from the multi‐center joint effort, it is still a time‐consuming process. In silico protein structure prediction has the potential to fill the gap and we will focus on the application of deep learning in the prediction of protein secondary and tertiary structures here.

Secondary structure refers to the regular local structure patterns that can usually be defined in three types, namely alpha helix, beta strand, and coiled coil. They can be further divided into a more detailed classification of 8 types.^[^ ^164^ ^]^ Secondary structure prediction is a residue‐level prediction problem and is usually aided by alignment of homologous sequences. The application of deep learning in secondary structure prediction has been reviewed recently.^[^ ^165^ ^]^ Use of a sliding window is a popular method to extract short to intermediate non‐local interactions, but architectures like CNN and BiLSTM can learn long‐range interactions through hierarchical representations. Applying different deep neural network architectures including hybrid networks has pushed the boundary of accuracy to around 85% for 3‐state (Q3) secondary structure prediction on the commonly used CB513 benchmark dataset.^[^ ^166^, ^167^, ^168^ ^]^ Recent research efforts focus more on 8‐state prediction (Q8), and most methods achieved overall accuracy above 70% on the CB513 dataset, with the maximum reported Q8 accuracy of 74% using ensembles.^[^ ^166^, ^169^, ^170^, ^171^ ^]^ It is worth pointing out that the performance in terms of accuracy and recall differs drastically for different secondary states.^[^ ^166^ ^]^ As expected, the most common alpha helix and beta strand states perform much better than others, while pi helices essentially cannot be predicted as the sample size is too small in training datasets. Future research is needed for improving the prediction accuracy of less common secondary structure states. Although the growing data in the PDB may help to alleviate the problem, better methods to handle the data imbalance are required.

Tertiary structure prediction commonly has two different approaches. For proteins whose homologs have known structures, they can be used as a template to jump‐start structure modeling since proteins with high sequence similarity also tend to show structure similarity (the same fold). Folding a protein in silico from scratch with physics or empirical energy potential assumes that a folded protein is at its native state with the lowest free energy. This is challenging because the search space is enormously large.^[^ ^162^ ^]^ Approaches like fragment assembly take advantage of existing peptides from PDB to help conformational sampling.^[^ ^172^ ^]^ Current state‐of‐the‐art methods to predict protein structures mostly utilize evolutionary information from a multiple sequence alignment (MSA), and ab initio folding with the first principles still seems far‐fetched.

Tertiary structure prediction has recently shown success in large protein families with co‐evolution methods.^[^ ^173^, ^174^ ^]^ Deep learning has further exploited co‐evolutionary information and significantly improved the prediction performance of protein structures without known homologous structures (free modeling or FM), which is highlighted in the breakthroughs in the latest round of Critical Assessment of protein Structure Prediction (CASP).^[^ ^175^ ^]^ CASP performance has entered a new era since CASP11, when residue‐residue contact predictions were introduced to assist structure modeling as constraints.^[^ ^176^ ^]^ Initially inferred from MSA by global statistics models,^[^ ^177^, ^178^, ^179^ ^]^ contact prediction has been found to be a suitable task for deep learning, especially CNN.^[^ ^180^, ^181^, ^182^ ^]^ CASP12 and the latest CASP13 have witnessed remarkable improvements, and the prominent success of AlphaFold last year raised considerable interest even from the general public.^[^ ^183^ ^]^ A major advancement in CASP13 is to predict distances in finer bins instead of binary contact, and highly accurate structure models were generated for a few targets from different groups.^[^ ^175^ ^]^

At the core of AlphaFold is a highly complex dilated residual neural network (ResNet) with 220 blocks to predict the C_β_ distances of residue pairs given the amino acid sequence and many MSA‐derived features.^[^ ^184^ ^]^ AlphaFold tried the more conventional fragment assembly approach to generate structure models initially in CASP13, but later found using gradient descent directly on the predicted protein‐specific potentials can produce similar results.^[^ ^185^ ^]^ The distance potential was first normalized with a universal reference distribution and then combined with backbone torsion angle distributions predicted with a similar neural network and also with potentials from Rosetta to prevent steric clashes.^[^ ^172^, ^186^ ^]^ Iterative optimization of the torsion angles based on the combined potential converged quickly to generate the backbone structure. Removing the reference potential subtraction or other terms slightly affected the performance, and further refining the structure model with a Rosetta relaxation protocol improved the accuracy slightly.^[^ ^184^ ^]^

AlphaFold ranked at the top overall, but for some targets, other groups were able to get the best models.^[^ ^175^ ^]^ The RaptorX software suite also predicts distances independently.^[^ ^187^ ^]^ The entry for contact and FM predictions, RaptorX‐Contact, ranked first in the contact prediction category of CASP13.^[^ ^188^ ^]^ Although AlphaFold did not submit contact predictions, it was reported to perform similarly.^[^ ^187^ ^]^ RaptorX‐Contact also used residual neural networks consisting of a 1D ResNet followed by a 2D ResNet, although the number of layers is much smaller compared to AlphaFold. Another major difference is in the folding pipeline, only the most likely distances are converted to constraints for Crystallography and NMR System (CNS) to fold the protein, which is a software for experimentally solving structures.^[^ ^189^ ^]^ Other top groups all seem to benefit from contact prediction via deep learning and used ResNet to predict the contact map from sequence, profile, and co‐evolutional information like the covariance matrix.^[^ ^190^, ^191^ ^]^ For example, Zhang's group ranked 1^st^, 3^rd^ and 5^th^ in template‐based modeling, for which targets are easier to predict and have homologs with known structure.^[^ ^192^ ^]^ They continued to improve their I‐TASSER and QUARK pipelines by carefully constructing MSA, integrating multiple contact prediction methods, and designing a new contact energy potential.^[^ ^190^ ^]^

**Figure 4:**
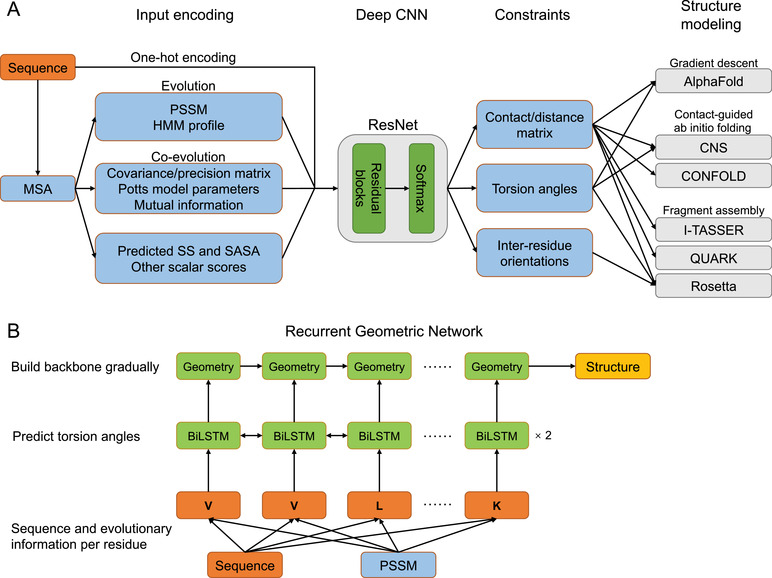
Workflow and network architectures of common protein structure prediction methods. A) Schematic summary of contact‐guided structure prediction methods. Different methods may use different kinds of features and network architectures, but co‐evolutionary information is essential for good contact prediction. Contact or distance between residue pairs and other predicted geometry constraints are fed into various methods for structure modelling or converted to protein‐specific potentials for direct optimization. SS, secondary structure; SASA, solvent accessible surface area. B) End‐to‐end recurrent geometric network predicts structure without co‐evolutionary information. First two BiLSTM layers predict backbone torsion angles and second a geometric layer adds residues one by one to construct the structure using torsion angles and atoms in the last residue.

The mainstream direction in the field of structure prediction now usually includes steps of MSA selection, contact/distance prediction, and structure modeling. The common workflow of popular methods is summarized in **Figure** **4**.^[^ ^184^, ^187^, ^190^, ^191^, ^193^, ^194^, ^195^, ^196^ ^]^ Using metagenomics databases and careful selection of deep MSA built from different algorithms and parameters help to obtain enough information to start.^[^ ^196^, ^197^ ^]^ Prediction of residue to residue geometry from co‐evolution by deep learning has proven to be crucial to limit the conformation search space. The latest advancement from Baker's group highlighted that predicting residue‐residue orientation together with distance in a multi‐task ResNet and integrating them in the Rosetta pipeline (trRosetta) outperformed top groups in CASP13.^[^ ^196^ ^]^ MSA selection and data augmentation via MSA sampling were shown to greatly contribute to the improvement as well. Finally, converting good geometry predictions to a good model is an important factor, and there are various approaches available (Figure 4A). For example, it was shown that when feeding predicted distances by Raptor‐Contact in CASP13 to trRosetta, the model accuracy increased significantly compared to CNS.^[^ ^196^ ^]^

Co‐evolution dependent methods dominated recent CASP experiments, partly due to the rapid growth of sequence databases. Although deep learning performs better on shallow MSA, very shallow MSA still poses a challenge for co‐evolution dependent methods.^[^ ^175^ ^]^ It is arguable that co‐evolution methods learn and construct a family average structure, which may have a resolution limit for functional insights.^[^ ^198^ ^]^ Recurrent geometric network (RGN) is a complementary method that just relies on primary sequence and position‐specific scoring matrices (PSSM) (evolutionary but not co‐evolutionary information) and uses RNN to build an end‐to‐end deep learning framework (Figure 4B).^[^ ^199^ ^]^ However, the local structure of predicted models may not be good enough and its performance in CASP13 is lower than the top groups. Another CNN‐based end‐to‐end method, NEMO, also only uses sequence and PSSM inputs and interestingly applies deep learning to the folding process.^[^ ^200^ ^]^ Ideally, the primary protein sequence should have all the information to fold a protein. Interestingly, structure predictions of designed proteins from one single sequence by trRosetta actually exhibited higher accuracy than naturally occurring proteins of similar sizes.^[^ ^196^ ^]^ The authors suggested that de novo proteins are ideal versions of natural proteins and the neural network can learn the general principle of protein structure. Structure prediction from very shallow MSA and even a single amino acid sequence remains a fundamental challenge, and the quality of models for larger proteins and proteins of multiple domains still has room for improvement.

## 9 Other Applications of Deep Learning in Proteomics

Besides the applications of deep learning described in the above sections, deep learning has also been used in many other sequence related applications in proteomics, including protein subcellular localization prediction,^[^ ^201^, ^202^ ^]^ protein‐protein interaction prediction,^[^ ^203^, ^204^ ^]^ protein function prediction,^[^ ^205^, ^206^ ^]^ peptide charge state distribution prediction,^[^ ^46^ ^]^ peptide detectability prediction,^[^ ^30^, ^210^ ^]^ and mutation impact on protein stability, function, and protein‐protein interaction.^[^ ^16^, ^207^, ^208^, ^209^ ^]^ Among these predictions, for example, charge state distribution prediction^[^ ^46^ ^]^ and peptide detectability prediction^[^ ^30^, ^210^ ^]^ have achieved highly accurate results using deep learning‐based methods with peptide sequences.

In addition to predicting peptide or protein properties using sequence data, deep learning has also been used to classify biological samples based on MS measurements in clinical proteomics. Kim et al.^[^ ^211^ ^]^ proposed a deep neural network‐based model to classify patients with pancreatic cancer using MRM‐MS data. The deep learning‐based model outperformed five traditional machine learning methods including RF and SVM. Dong et al.^[^ ^212^ ^]^ developed a CNN‐based model to discriminate tumor from normal samples. The model uses precursors and their extracted ion chromatograms from raw DDA MS/MS data as input. Because this method does not require peptide or protein identification and quantification, many precursors that may not be identified in a typical protein identification workflow could be used in the modeling and thereby contribute to the classification. The performance of this model was shown to be superior to four traditional machine learning methods including SVM, RF, and gradient boosting decision tree on three large‐scale public datasets. More recently, Zhang et al.^[^ ^213^ ^]^ proposed an MS data representation method called DIA tensor and developed a deep neural network (ResNet) to work with DIA tensor for phenotype prediction on DIA‐MS data. Similar to the Dong et al. study,^[^ ^212^ ^]^ this method does not require protein identification and quantification either. The performance of this method was demonstrated on two large scale DIA datasets. Despite these exciting developments, application of deep learning to biological sample classification is typically limited by the sample size of clinical cohorts. Moreover, such application has a higher requirement on model interpretability than other applications described in the paper.

## 10 Conclusion and Perspectives

Deep learning has great potential in many areas of proteomics research. With continuous improvements to deep learning techniques and generation of high‐quality proteomics data, we expect deep learning will have a profound impact in the application areas reviewed in this study and beyond. It may revolutionize how we analyze proteomics data in the near future.

Although deep learning has been highly successful in predicting many peptide or protein properties, some properties are still difficult to predict. Predicting RT and MS/MS spectrum for peptides with complicated modifications like glycosylation remains challenging. There is clearly room for improvement for deep learning‐based peptide de novo sequencing. For cross linked peptides, there is no published deep learning‐based tool for either RT or MS/MS spectrum prediction. For PTMs with limited or no known sites, deep learning‐based prediction is almost impossible. For many MHC class I and II alleles with limited numbers of known binding peptides, there is still large room for improvement. Another interesting topic without any published deep learning‐based tools is the relationship prediction between actual peptide amount and peptide intensity in mass spectrometry experiments. Because the signal response in MS for different peptides is different, the abundance levels generated using MS for different peptides are not directly comparable. Thus, it is almost impossible to directly estimate absolute quantification for proteins from MS data with similar accuracy to RNA‐Seq for gene quantification. However, if sufficient numbers of proteins with known actual amount and their abundance data from mass spectrometry are available, it may be possible to train a model for proteome wide absolute protein quantification with high accuracy. This may open the door to a lot of applications.

The generalizability of models is an important consideration in model development and application. Both the RT and MS/MS spectrum of a peptide are highly associated with experiment conditions. The RT and MS/MS spectrum prediction are typically associated with a specific experiment setting. For example, a small change to the LC conditions (e.g., pore size, column material, column setup, dead volume, sample loading, etc.) may lead to a drastic change of the RT of a peptide. Therefore, an RT model trained using the data from one experiment may have large prediction error when applied to another experiment with different LC conditions. Therefore, it would be difficult to develop a generic predictor that could be applied to multiple experiments with different LC settings. One solution for deriving a generic model is to use data based on indexed retention time (iRT)^[^ ^214^ ^]^; however, this requires adding iRT peptides in all experiments. MS/MS spectrum prediction is less affected by experimental settings compared to RT prediction. In general, only the type of MS instrument, peptide fragmentation method, and collision energy require consideration in MS/MS spectrum prediction. A generic model could be developed by considering these factors in the model training as implemented in pDeep2. Because changes in any of these conditions may alter the spectrum pattern of a peptide, these conditions also need to be considered in deep learning‐based de novo peptide sequencing during both model training and application. In addition, peptide sequence patterns could be learned during model training in deep learning‐based de novo peptide sequencing, but different species may have different sequence patterns. Thus, species is another factor for consideration in model training and application. A generic model may be trained by considering all these factors in an efficient way. Other predictions, including PTM site prediction, MHC‐binding prediction, and protein structure prediction, are typically not associated with a specific experiment. These predictors tend to be generalizable. Public data repositories^[^ ^135^, ^215^, ^216^ ^]^ are valuable resources of training data for most of these predictors. Comprehensive meta data for these pubic data sets is critical for data reuse.

In general, evaluation accompanies each tool to demonstrate its performance by comparing with other similar tools. In many studies, pre‐trained models from previous studies were used for comparison. This type of comparison is likely to be biased depending on the training data. Sometimes it is difficult to train the models using the same training data from scratch due to a variety of reasons. In many cases the tool cannot be retrained due to the availability of source code or lack enough documentation in the original publications for researchers to reproduce the training method using user provided data. Even so, independent comprehensive evaluation of the performance of these deep learning tools is critical to provide guidance to users for method selection since one tool may have significantly different performance on different datasets or using different evaluation metrics. Rigorous documentation of methods for training in addition to functions for retraining models when tools are published would benefit the independent evaluation process.

Some applications are limited by the size of training data. A close collaboration between data scientists and experimentalists could help generate appropriate experimental datasets for model training. Technically, transfer learning and semi‐supervised learning can also be used to partially overcome the problem of small training data. In addition, both proteomics data and the outcome variables may be noisy for some applications. Designing deep learning models that are robust to noise in the training data would be particularly useful.

An active research direction focuses on novel representation methods for protein sequence data. Recent studies show that models based on natural language processing inspired techniques such as Transformer,^[^ ^217^ ^]^ BERT,^[^ ^218^ ^]^ and GPT‐2^[^ ^219^ ^]^ can learn features from a large corpus of protein sequences in a self‐supervised fashion, with applications in a variety of downstream tasks.^[^ ^220^, ^221^ ^]^ Besides a linear sequence of amino acids, proteins can also be modeled as a graph to capture both structure and sequence information. Graph neural networks^[^ ^222^ ^]^ are powerful deep learning architectures for learning representations of nodes and edges from such data.^[^ ^223^ ^]^

Another promising direction is the use of NAS to aid the design of deep learning models. Developing a high performing deep neural network requires significant architecture engineering including the selection of a basic neural network architecture (such as CNN, RNN, or combination of them) and hyperparameter tuning. However, due to the huge search space, without using carefully designed neural architecture search algorithms, it is generally very time‐consuming and inefficient and also requires extensive knowledge about deep learning to manually design a high performance model. NAS has been demonstrated to be a powerful approach to the design of neural architectures in many other research areas.^[^ ^49^, ^50^ ^]^ We expect this technique will have a broader application in proteomics in the future.

Despite superior performance, deep learning models are typically considered to be black‐boxes because how the models make the prediction and what the models learn from the input data are largely unknown. Interpretability in deep learning is still a big challenge. Different algorithms and tools have been developed to tackle this challenge, such as algorithms including integrated gradients^[^ ^79^ ^]^ and tools including Captum (https://captum.ai/). However, few of them have been applied to deep learning applications in proteomics. Adoption of these algorithms will help researchers better understand how the deep learning models work and will provide new insights into the mechanisms underlying the proteomic problems under investigation.

## Conflict of Interest

The authors declare no conflict of interest.

## References

[1] P. Kelchtermans , W. Bittremieux , K. De Grave , S. Degroeve , J. Ramon , K. Laukens , D. Valkenborg , H. Barsnes , L. Martens , Proteomics 2014, 14, 353. 24323524 10.1002/pmic.201300289

[2] R. Bouwmeester , R. Gabriels , T. Van Den Bossche , L. Martens , S. Degroeve , Proteomics 2020, e1900351. 32267083 10.1002/pmic.201900351

[3] L. L. Xu , A. Young , A. Zhou , H. L. Rost , Proteomics 2020, e1900352. 32061181 10.1002/pmic.201900352

[4] T. Ching , D. S. Himmelstein , B. K. Beaulieu‐Jones , A. A. Kalinin , B. T. Do , G. P. Way , E. Ferrero , P.‐M. Agapow , M. Zietz , M. M. Hoffman , W. Xie , G. L. Rosen , B. J. Lengerich , J. Israeli , J. Lanchantin , S. Woloszynek , A. E. Carpenter , A. Shrikumar , J. Xu , E. M. Cofer , C. A. Lavender , S. C. Turaga , A. M. Alexandari , Z. Lu , D. J. Harris , D. DeCaprio , Y. Qi , A. Kundaje , Y. Peng , L. K. Wiley , M. H. S. Segler , S. M. Boca , S. J. Swamidass , A. Huang , A. Gitter , C. S. Greene , J. R. Soc. Interface. 2018, 15, 20170387. 29618526 10.1098/rsif.2017.0387 PMC5938574

[5] C. Cao , F. Liu , H. Tan , D. Song , W. Shu , W. Li , Y. Zhou , X. Bo , Z. Xie , Genomics Proteomics Bioinf. 2018, 16, 17. 10.1016/j.gpb.2017.07.003 PMC6000200 29522900

[6] R. Dias , A. Torkamani , Genome. Med. 2019, 11, 70. 31744524 10.1186/s13073-019-0689-8 PMC6865045

[7] S. Min , B. Lee , S. Yoon , Brief. Bioinform. 2017, 18, 851. 27473064 10.1093/bib/bbw068

[8] G. Eraslan , Z. Avsec , J. Gagneur , F. J. Theis , Nat. Rev. Genet. 2019, 20, 389. 30971806 10.1038/s41576-019-0122-6

[9] I. Goodfellow , Y. Bengio , A. Courville , Deep Learning, The MIT Press, Cambridge, MA 2016.

[10] N. H. Tran , X. Zhang , L. Xin , B. Shan , M. Li , Proc. Natl. Acad. Sci. USA 2017, 114, 8247. 28720701 10.1073/pnas.1705691114 PMC5547637

[11] S. Henikoff , J. G. Henikoff , Proc. Natl. Acad. Sci. USA 1992, 89, 10915. 1438297 10.1073/pnas.89.22.10915 PMC50453

[12] E. Asgari , M. R. Mofrad , PLoS One 2015, 10, e0141287. 26555596 10.1371/journal.pone.0141287 PMC4640716

[13] K. K. Yang , Z. Wu , C. N. Bedbrook , F. H. Arnold , Bioinformatics 2018, 34, 2642. 29584811 10.1093/bioinformatics/bty178 PMC6061698

[14] H. ElAbd , Y. Bromberg , A. Hoarfrost , T. Lenz , A. Franke , M. Wendorff , BMC Bioinf. 2020, 21, 235. 10.1186/s12859-020-03546-x PMC7285590 32517697

[15] N. Strodthoff , P. Wagner , M. Wenzel , W. Samek , Bioinformatics 2020, 36, 2401. 31913448 10.1093/bioinformatics/btaa003 PMC7178389

[16] E. C. Alley , G. Khimulya , S. Biswas , M. AlQuraishi , G. M. Church , Nat. Methods 2019, 16, 1315. 31636460 10.1038/s41592-019-0598-1 PMC7067682

[17] Y. LeCun , Y. Bengio , G. Hinton , Nature 2015, 521, 436. 26017442 10.1038/nature14539

[18] S. Hochreiter , J. Schmidhuber , Neural. Comput. 1997, 9, 1735. 9377276 10.1162/neco.1997.9.8.1735

[19] J. Chung , C. Gulcehre , K. Cho , Y. Bengio , arXiv preprint, arXiv:1412.3555, 2014.

[20] S. Sabour , N. Frosst , G. E. Hinton , Advances in Neural Information Processing Systems 2017, 3856.

[21] V. Dorfer , S. Maltsev , S. Winkler , K. Mechtler , R. T. Charme , J. Proteome. Res. 2018, 17, 2581. 29863353 10.1021/acs.jproteome.7b00836 PMC6079931

[22] A. T. Chen , A. Franks , N. Slavov , PLoS Comput. Biol. 2019, 15, e1007082. 31260443 10.1371/journal.pcbi.1007082 PMC6625733

[23] E. F. Strittmatter , L. J. Kangas , K. Petritis , H. M. Mottaz , G. A. Anderson , Y. Shen , J. M. Jacobs , D. G. Camp 2nd , R. D. Smith , J. Proteome. Res. 2004, 3, 760. 15359729 10.1021/pr049965y

[24] A. A. Klammer , X. Yi , M. J. MacCoss , W. S. Noble , Anal. Chem. 2007, 79, 6111. 17622186 10.1021/ac070262k

[25] B. Wen , K. Li , Y. Zhang , B. Zhang , Nat. Commun. 2020, 11, 1759. 32273506 10.1038/s41467-020-15456-w PMC7145864

[26] B. Blank‐Landeshammer , I. Teichert , R. Marker , M. Nowrousian , U. Kück , A. Sickmann , mBio 2019, 10, e02367. 31615963 10.1128/mBio.02367-19 PMC6794485

[27] E. Lau , Y. Han , D. R. Williams , C. T. Thomas , R. Shrestha , J. C. Wu , M. P. Y. Lam , Cell Rep. 2019, 29, 3751. 31825849 10.1016/j.celrep.2019.11.026 PMC6961840

[28] T. Ouspenskaia , T. Law , K. R. Clauser , S. Klaeger , S. Sarkizova , F. Aguet , B. Li , E. Christian , B. A. Knisbacher , P. M. Le , C. R. Hartigan , H. Keshishian , A. Apffel , G. Oliveira , W. Zhang , Y. T. Chow , Z. Ji , S. A. Shukla , P. Bachireddy , G. Getz , N. Hacohen , D. B. Keskin , S. A. Carr , C. J. Wu , A. Regev , *bioRxiv* 2020 10.1101/2020.02.12.945840

[29] S. Gessulat , T. Schmidt , D. P. Zolg , P. Samaras , K. Schnatbaum , J. Zerweck , T. Knaute , J. Rechenberger , B. Delanghe , A. Huhmer , U. Reimer , H.‐C. Ehrlich , S. Aiche , B. Kuster , M. Wilhelm , Nat. Methods. 2019, 16, 509. 31133760 10.1038/s41592-019-0426-7

[30] Y. Yang , X. Liu , C. Shen , Y. Lin , P. Yang , L. Qiao , Nat. Commun. 2020, 11, 146. 31919359 10.1038/s41467-019-13866-z PMC6952453

[31] R. Lou , P. Tang , K. Ding , S. Li , C. Tian , Y. Li , S. Zhao , Y. Zhang , W. Shui , iScience 2020, 23, 100903. 32109675 10.1016/j.isci.2020.100903 PMC7044796

[32] B. C. Searle , K. E. Swearingen , C. A. Barnes , T. Schmidt , S. Gessulat , B. Küster , M. Wilhelm , Nat. Commun. 2020, 11, 1548. 32214105 10.1038/s41467-020-15346-1 PMC7096433

[33] B. Van Puyvelde , S. Willems , R. Gabriels , S. Daled , L. De Clerck , S. V. Casteele , A. Staes , F. Impens , D. Deforce , L. Martens , S. Degroeve , M. Dhaenens , Proteomics 2020, 20, e1900306. 31981311 10.1002/pmic.201900306

[34] J. L. Meek , Proc. Natl. Acad. Sci. U S A. 1980, 77, 1632. 6929513 10.1073/pnas.77.3.1632 PMC348551

[35] D. Guo , C. T. Mant , A. K. Taneja , J. M. R. Parker , R. S. Rodges , J. Chromatogr. A 1986, 359, 499.

[36] D. Gussakovsky , H. Neustaeter , V. Spicer , O. V. Krokhin , Anal. Chem. 2017, 89, 11795. 28971681 10.1021/acs.analchem.7b03436

[37] W. Lu , X. Liu , S. Liu , W. Cao , Y. Zhang , P. Yang , Sci. Rep. 2017, 7, 43959. 28303880 10.1038/srep43959 PMC5356008

[38] H. Maboudi Afkham , X. Qiu , M. The , L. Kall , Bioinformatics 2017, 33, 508. 27797755 10.1093/bioinformatics/btw619

[39] K. Petritis , L. J. Kangas , P. L. Ferguson , G. A. Anderson , L. Pasa‐Tolic , M. S. Lipton , K. J. Auberry , E. F. Strittmatter , Y. Shen , R. Zhao , R. D. Smith , Anal. Chem. 2003, 75, 1039. 12641221 10.1021/ac0205154

[40] O. V. Krokhin , R. Craig , V. Spicer , W. Ens , K. G. Standing , R. C. Beavis , J. A. Wilkins , Mol. Cell. Proteomics. 2004, 3, 908. 15238601 10.1074/mcp.M400031-MCP200

[41] O. V. Krokhin , Anal. Chem. 2006, 78, 7785. 17105172 10.1021/ac060777w

[42] L. Moruz , D. Tomazela , L. Kall , J. Proteome. Res. 2010, 9, 5209. 20735070 10.1021/pr1005058

[43] L. Moruz , A. Staes , J. M. Foster , M. Hatzou , E. Timmerman , L. Martens , L. Käll , Proteomics 2012, 12, 1151. 22577017 10.1002/pmic.201100386

[44] C. Ma , Y. Ren , J. Yang , Z. Ren , H. Yang , S. Liu , Anal. Chem. 2018, 90, 10881. 30114359 10.1021/acs.analchem.8b02386

[45] S. Tiwary , R. Levy , P. Gutenbrunner , F. Salinas Soto , K. K. Palaniappan , L. Deming , M. Berndl , A. Brant , P. Cimermancic , J. Cox , Nat. Methods. 2019, 16, 519. 31133761 10.1038/s41592-019-0427-6

[46] S. Guan , M. F. Moran , B. Ma , Mol. Cell. Proteomics. 2019, 18, 2099. 31249099 10.1074/mcp.TIR119.001412 PMC6773555

[47] R. Bouwmeester , R. Gabriels , N. Hulstaert , L. Martens , S. Degroeve , bioRxiv 2020 10.1101/2020.03.28.013003 34711972

[48] D. Bahdanau , K. Cho , Y. Bengio , Neural Machine Translation by Jointly Learning to Align and Translate, arXiv:1409.0473, 2014.

[49] T. Elsken , J. H. Metzen , F. Hutter , J. Mach. Learn. Res. 2019, 20, 1.

[50] K. O. Stanley , J. Clune , J. Lehman , R. Miikkulainen , Nat. Mach. Intell. 2019, 1, 24.

[51] K. Li , A. Jain , A. Malovannaya , B. Wen , B. Zhang , Proteomics 2020, e1900334. 32864883 10.1002/pmic.201900334 PMC7718998

[52] Z. Noor , S. B. Ahn , M. S. Baker , S. Ranganathan , A. Mohamedali , Brief. Bioinform. 2020, bbz163. 32047889 10.1093/bib/bbz163

[53] S. J. Barton , J. C. Whittaker , Mass. Spectrom. Rev. 2009, 28, 177. 18680189 10.1002/mas.20188

[54] J. K. Eng , A. L. McCormack , J. R. Yates , J. Am. Soc. Mass. Spectrom. 1994, 5, 976. 24226387 10.1016/1044-0305(94)80016-2

[55] S. Li , R. J. Arnold , H. Tang , P. Radivojac , Anal. Chem. 2011, 83, 790. 21175207 10.1021/ac102272r PMC3036742

[56] K. Liu , S. Li , L. Wang , Y. Ye , H. Tang , Anal. Chem. 2020, 92, 4275. 32053352 10.1021/acs.analchem.9b04867 PMC8057055

[57] X. X. Zhou , W. F. Zeng , H. Chi , C. Luo , J. Zhan , S.‐M. He , Z. Zhang , Anal. Chem. 2017, 89, 12690. 29125736 10.1021/acs.analchem.7b02566

[58] Z. Zhang , Anal. Chem. 2004, 76, 3908. 15253624 10.1021/ac049951b

[59] Z. Zhang , Anal. Chem. 2005, 77, 6364. 16194101 10.1021/ac050857k

[60] Z. Zhang , Anal. Chem. 2011, 83, 8642. 21995278 10.1021/ac2020917

[61] Y. Wang , F. Yang , P. Wu , D. Bu , S. Sun , BMC Bioinformatics 2015, 16, 110. 25887925 10.1186/s12859-015-0540-1 PMC4415337

[62] R. J. Arnold , N. Jayasankar , D. Aggarwal , H. Tang , P. Radivojac , Pac. Symp. Biocomput. 2006, 219. 17094241

[63] S. Degroeve , L. Martens , Bioinformatics 2013, 29, 3199. 24078703 10.1093/bioinformatics/btt544 PMC5994937

[64] S. Degroeve , D. Maddelein , L. Martens , Nucleic. Acids. Res. 2015, 43, W326. 25990723 10.1093/nar/gkv542 PMC4489309

[65] R. Gabriels , L. Martens , S. Degroeve , Nucleic. Acids. Res. 2019, 47, W295. 31028400 10.1093/nar/gkz299 PMC6602496

[66] N. P. Dong , Y. Z. Liang , Q. S. Xu , D. K. Mok , L.‐Z. Yi , H.‐M. Lu , M. He , W. Fan , Anal. Chem. 2014, 86, 7446. 25032905 10.1021/ac501094m

[67] C. Zhou , L. D. Bowler , J. Feng , BMC Bioinformatics 2008, 9, 325. 18664292 10.1186/1471-2105-9-325 PMC2529326

[68] A. M. Frank , J. Proteome. Res. 2009, 8, 2226. 19256476 10.1021/pr800677f PMC2738854

[69] W. F. Zeng , X. X. Zhou , W. J. Zhou , H. Chi , J. Zhan , S.‐M. He , Anal. Chem. 2019, 91, 9724. 31283184 10.1021/acs.analchem.9b01262

[70] Y. M. Lin , C. T. Chen , J. M. Chang , BMC Genomics 2019, 20, 906. 31874640 10.1186/s12864-019-6297-6 PMC6929458

[71] Y. Lecun , L. Bottou , Y. Bengio , P. Haffner , Proc. IEEE 1998, 86, 2278.

[72] A. Michalski , N. Neuhauser , J. Cox , M. Mann , J. Proteome. Res. 2012, 11, 5479. 22998608 10.1021/pr3007045

[73] W. J. Zhou , H. Yang , W. F. Zeng , K. Zhang , H. Chi , S.‐M. He , J. Proteome. Res. 2019, 18, 2747. 31244209 10.1021/acs.jproteome.8b00993

[74] H. Yang , H. Chi , W. F. Zeng , W. J. Zhou , S. M. He , Bioinformatics 2019, 35, i183. 31510687 10.1093/bioinformatics/btz366 PMC6612832

[75] G. Rosenberger , C. C. Koh , T. Guo , H. L. Rost , P. Kouvonen , B. C. Collins , M. Heusel , Y. Liu , E. Caron , A. Vichalkovski , M. Faini , O. T. Schubert , P. Faridi , H. A. Ebhardt , M. Matondo , H. Lam , S. L. Bader , D. S. Campbell , E. W. Deutsch , R. L. Moritz , S. Tate , R. Aebersold , Sci. Data. 2014, 1, 140031. 25977788 10.1038/sdata.2014.31 PMC4322573

[76] J. Zi , S. Zhang , R. Zhou , B. Zhou , S. Xu , G. Hou , F. Tan , B. Wen , Q. Wang , L. Lin , S. Liu , Anal. Chem. 2014, 86, 7242. 24969961 10.1021/ac501828a

[77] T. Zhu , Y. Zhu , Y. Xuan , H. Gao , X. Cai , S. R. Piersma , T. V. Pham , T. Schelfhorst , R. R. G. D. Haas , I. V., Bijnsdorp , R. Sun , L. Yue , G. Ruan , Q. Zhang , M. Hu , Y. Zhou , W. J. Van Houdt , T. Y. S., Le Large , J. Cloos , A. Wojtuszkiewicz , D. Koppers‐Lalic , F. Böttger , C. Scheepbouwer , R. H. Brakenhoff , G. J. L. H. van Leenders , J. N. M. Ijzermans , J. W. M. Martens , R. D. M. Steenbergen , N. C. Grieken , S. Selvarajan , S. Mantoo , S. S. Lee , S. J. Y. Yeow , S. M. F. Alkaff , N. Xiang , Y. Sun , X. Yi , S. Dai , W. Liu , T. Lu , Z. Wu , X. Liang , M. Wang , Y. Shao , X. Zheng , K. Xu , Q. Yang , Y. Meng , C. Lu , J. Zhu , J. Zheng , B. Wang , S. Lou , Y. Dai , C. Xu , C. Yu , H. Ying , T. K. Lim , J. Wu , X. Gao , Z. Luan , X. Teng , P. Wu , S. Huang , Z. Tao , N. G. Iyer , S. Zhou , W. Shao , H. Lam , D. Ma , J. Ji , O. L. Kon , S. Zheng , R. Aebersold , C. R. Jimenez , T. Guo , Genom. Proteom. Bioinf. 2020, S1672.

[78] J. Doellinger , C. Blumenscheit , A. Schneider , P. Lasch , Anal. Chem. 2020, 92, 12185. 32840101 10.1021/acs.analchem.0c00994

[79] M. Sundararajan , A. Taly , Q. Yan , *arXiv:1703.01365* , 2017.

[80] R. Xu , J. Sheng , M. Bai , K. Shu , Y. Zhu , C. Chang , Proteomics 2020, e1900345. 32574431 10.1002/pmic.201900345

[81] O. Vinyals , A. Toshev , S. Bengio , D. Erhan , 2015 IEEE Conf. on Computer Vision and Pattern Recognition (CVPR), IEEE, Piscataway, NJ 2015, pp. 3156–3164.

[82] A. Karpathy , L. Fei‐Fei , IEEE Trans. Pattern. Anal. Mach. Intell. 2017, 39, 664. 27514036 10.1109/TPAMI.2016.2598339

[83] B. Ma , K. Zhang , C. Hendrie , C. Liang , M. Li , A. Doherty‐Kirby , G. Lajoie , Rapid. Commun. Mass. Spectrom. 2003, 17, 2337. 14558135 10.1002/rcm.1196

[84] B. Ma , J. Am. Soc. Mass. Spectrom. 2015, 26, 1885. 26122521 10.1007/s13361-015-1204-0 PMC4604512

[85] A. Frank , P. Pevzner , Anal. Chem. 2005, 77, 964. 15858974 10.1021/ac048788h

[86] N. H. Tran , R. Qiao , L. Xin , X. Chen , C. Liu , X. Zhang , B. Shan , A. Ghodsi , M. Li , Nat. Methods 2019, 16, 63. 30573815 10.1038/s41592-018-0260-3

[87] K. Karunratanakul , H. Y. Tang , D. W. Speicher , E. Chuangsuwanich , S. Sriswasdi , Mol. Cell. Proteomics. 2019, 18, 2478. 31591261 10.1074/mcp.TIR119.001656 PMC6885704

[88] T. Muth , B. Y. Renard , Brief. Bioinform. 2018, 19, 954. 28369237 10.1093/bib/bbx033

[89] H. Chi , H. Chen , K. He , L. Wu , B. Yang , R.‐X. Sun , J. Liu , W.‐F. Zeng , C.‐Q. Song , S.‐M. He , M.‐Q. Dong , J. Proteome. Res. 2013, 12, 615. 23272783 10.1021/pr3006843

[90] Y. A. Qi , T. K. Maity , C. M. Cultraro , V. Misra , X. Zhang , C. Ade , S. Gao , D. Milewski , K. D. Nguyen , M. H. Ebrahimabadi , K.‐I. Hanada , J. Khan , C. Sahinalp , J. C. Yang , U. Guha , *bioRxiv* 2020 10.1101/2020.08.04.236331 PMC8724932 34391887

[91] J.‐Y. Lee , H. D. Mitchell , M. C. Burnet , S. C. Jenson , E. D. Merkley , A. K. Shukla , E. S. Nakayasu , S. H. Payne , bioRxiv 2018 10.1101/428334

[92] E. S. Witze , W. M. Old , K. A. Resing , N. G. Ahn , Nat. Methods. 2007, 4, 798. 17901869 10.1038/nmeth1100

[93] Y. C. Wang , S. E. Peterson , J. F. Loring , Cell. Res. 2014, 24, 143. 24217768 10.1038/cr.2013.151 PMC3915910

[94] A. H. Millar , J. L. Heazlewood , C. Giglione , M. J. Holdsworth , A. Bachmair , W. X. Schulze , Annu. Rev. Plant. Biol. 2019, 70, 119. 30786234 10.1146/annurev-arplant-050718-100211

[95] Y. Zhao , O. N. Jensen , Proteomics 2009, 9, 4632. 19743430 10.1002/pmic.200900398 PMC2892724

[96] D. Wang , S. Zeng , C. Xu , W. Qiu , Y. Liang , T. Joshi , D. Xu , Bioinformatics 2017, 33, 3909. 29036382 10.1093/bioinformatics/btx496 PMC5860086

[97] F. Luo , M. Wang , Y. Liu , X. M. Zhao , A. Li , Bioinformatics 2019, 35, 2766. 30601936 10.1093/bioinformatics/bty1051 PMC6691328

[98] E. Fenoy , J. M. G. Izarzugaza , V. Jurtz , S. Brunak , M. Nielsen , Bioinformatics 2019, 35, 1098. 30169744 10.1093/bioinformatics/bty715

[99] K. E. Kirchoff , S. M. Gomez , *bioRxiv* 2020 10.1101/2020.02.04.934216

[100] I. Deznabi , B. Arabaci , M. Koyuturk , O. Tastan , Bioinformatics 2020, 36, 3652. 32044914 10.1093/bioinformatics/btaa013 PMC7320620

[101] H. Fu , Y. Yang , X. Wang , H. Wang , Y. Xu , BMC Bioinformatics 2019, 20, 86. 30777029 10.1186/s12859-019-2677-9 PMC6379983

[102] F. He , R. Wang , J. Li , L. Bao , D. Xu , X. Zhao , BMC Syst. Biol. 2018, 12, 109. 30463553 10.1186/s12918-018-0628-0 PMC6249717

[103] X. Zhao , J. Li , R. Wang , F. He , L. Yue , M. Yin , IEEE Access 2018, 6, 63560.

[104] K. Yu , Q. Zhang , Z. Liu , Y. Du , X. Gao , Q. Zhao , H. Cheng , X. Li , Z.‐X. Liu , Brief. Bioinform. 2019, bbz107, 10.1093/bib/bbz107 32978618

[105] B. Yu , Z. Yu , C. Chen , A. Ma , B. Liu , B. Tian , Q. Ma , Chemom. Intell. Lab. Syst. 2020, 200, 103999.

[106] M. Wu , Y. Yang , H. Wang , Y. Xu , BMC Bioinformatics 2019, 20, 49. 30674277 10.1186/s12859-019-2632-9 PMC6343287

[107] G. Taherzadeh , A. Dehzangi , M. Golchin , Y. Zhou , M. P. Campbell , Bioinformatics 2019, 35, 4140. 30903686 10.1093/bioinformatics/btz215

[108] Z. Chen , N. He , Y. Huang , W. T. Qin , X. Liu , L. Li , Genom. Proteom. Bioinf. 2018, 16, 451. 10.1016/j.gpb.2018.08.004 PMC6411950 30639696

[109] J. Sun , Y. Cao , D. Wang , W. Bao , Y. Chen , IEEE Access 2020, 8, 47304.

[110] N. Thapa , M. Chaudhari , S. McManus , K. Roy , R. H. Newman , H. Saigo , D. B. Kc , BMC Bioinformatics 2020, 21, 63. 32321437 10.1186/s12859-020-3342-z PMC7178942

[111] K. Y. Huang , J. B. Hsu , T. Y. Lee , Sci. Rep. 2019, 9, 16175. 31700141 10.1038/s41598-019-52552-4 PMC6838336

[112] J. Chen , R. Yang , C. Zhang , L. Zhang , Q. Zhang , IEEE Access 2019, 7, 142368.

[113] Y. Xie , X. Luo , Y. Li , L. Chen , W. Ma , J. Huang , J. Cui , Y. Zhao , Y. Xue , Z. Zuo , J. Ren , Genom. Proteom. Bioinf. 2018, 16, 294. 10.1016/j.gpb.2018.04.007 PMC6205083 30268931

[114] Y. Zhao , N. He , Z. Chen , L. Li , IEEE Access 2020, 8, 14244.

[115] H. Long , B. Liao , X. Xu , J. Yang , Int. J. Mol. Sci. 2018, 19, 2817. 10.3390/ijms19092817 PMC6164125 30231550

[116] W. Ning , P. Jiang , Y. Guo , C. Wang , X. Tan , W. Zhang , D. Peng , Y. Xue , Brief. Bioinform. 2020, bbaa038. 32248222 10.1093/bib/bbaa038

[117] D. Wang , Y. Liang , D. Xu , Bioinformatics 2019, 35, 2386. 30520972 10.1093/bioinformatics/bty977 PMC6612812

[118] J. Gao , J. J. Thelen , A. K. Dunker , D. Xu , Mol. Cell. Proteomics. 2010, 9, 2586. 20702892 10.1074/mcp.M110.001388 PMC3101956

[119] N. Blom , T. Sicheritz‐Ponten , R. Gupta , S. Gammeltoft , S. Brunak , Proteomics 2004, 4, 1633. 15174133 10.1002/pmic.200300771

[120] V. Pejaver , W. L. Hsu , F. Xin , A. K. Dunker , V. N. Uversky , P. Radivojac , Protein. Sci. 2014, 23, 1077. 24888500 10.1002/pro.2494 PMC4116656

[121] L. Wei , P. Xing , J. Tang , Q. Zou , IEEE Trans. Nanobiosci. 2017, 16, 240. 10.1109/TNB.2017.2661756 28166503

[122] Y. Xue , J. Ren , X. Gao , C. Jin , L. Wen , X. Yao , Mol. Cell. Proteomics. 2008, 7, 1598. 18463090 10.1074/mcp.M700574-MCP200 PMC2528073

[123] Y. Xue , Z. Liu , J. Cao , Q. Ma , X. Gao , Q. Wang , C. Jin , Y. Zhou , L. Wen , J. Ren , Protein. Eng. Des. Sel. 2011, 24, 255. 21062758 10.1093/protein/gzq094

[124] D. Chicco , Methods. Mol. Biol. 2021, 2190, 73. 32804361 10.1007/978-1-0716-0826-5_3

[125] S. R. Savage , B. Zhang , Clin. Proteomics 2020, 17, 27. 32676006 10.1186/s12014-020-09290-x PMC7353784

[126] E. J. Needham , B. L. Parker , T. Burykin , D. E. James , S. J. Humphrey , Sci. Signal 2019, 12, eaau8645. 30670635 10.1126/scisignal.aau8645

[127] Y. Xian , B. Schiele , Z. Akata , Proc. of the IEEE Conf. on Computer Vision and Pattern Recognition, IEEE, Piscataway, NJ 2017, pp. 4582–4591.

[128] D. Wang , D. Liu , J. Yuchi , F. He , Y. Jiang , S. Cai , J. Li , D. Xu , Nucleic. Acids. Res. 2020, 48, W140. 32324217 10.1093/nar/gkaa275 PMC7319475

[129] M. Mann , O. N. Jensen , Nat. Biotechnol. 2003, 21, 255. 12610572 10.1038/nbt0303-255

[130] D. Ochoa , A. F. Jarnuczak , C. Vieitez , M. Gehre , M. Soucheray , A. Mateus , A. A. Kleefeldt , A. Hill , L. Garcia‐Alonso , F. Stein , N. J. Krogan , M. M. Savitski , D. L. Swaney , J. A. Vizcaíno , K.‐M. Noh , P. Beltrao , Nat. Biotechnol. 2020, 38, 365. 31819260 10.1038/s41587-019-0344-3 PMC7100915

[131] J. Neefjes , M. L. Jongsma , P. Paul , O. Bakke , Nat. Rev. Immunol. 2011, 11, 823. 22076556 10.1038/nri3084

[132] P. E. Jensen , Nat. Immunol. 2007, 8, 1041. 17878914 10.1038/ni1516

[133] D. Gfeller , M. Bassani‐Sternberg , Front. Immunol. 2018, 9, 1716. 30090105 10.3389/fimmu.2018.01716 PMC6068240

[134] Y. Kim , J. Ponomarenko , Z. Zhu , D. Tamang , P. Wang , J. Greenbaum , C. Lundegaard , A. Sette , O. Lund , P. E. Bourne , M. Nielsen , B. Peters , Nucleic. Acids. Res. 2012, 40, W525. 22610854 10.1093/nar/gks438 PMC3394288

[135] W. Shao , P. G. A. Pedrioli , W. Wolski , C. Scurtescu , E. Schmid , J. A. Vizcaíno , M. Courcelles , H. Schuster , D. Kowalewski , F. Marino , C. S. L. Arlehamn , K. Vaughan , B. Peters , A. Sette , T. H. M. Ottenhoff , K. E. Meijgaarden , N. Nieuwenhuizen , S. H. E. Kaufmann , R. Schlapbach , J. C. Castle , A. I. Nesvizhskii , M. Nielsen , E. W. Deutsch , D. S. Campbell , R. L. Moritz , R. A. Zubarev , A. J. Ytterberg , A. W. Purcell , M. Marcilla , A. Paradela , Q. Wang , C. E. Costello , N. Ternette , P. A. van Veelen , C. A. C. M. van Els , A. J. R. Heck , G. A. de Souza , L. M. Sollid , A. Admon , S. Stevanovic , H.‐G. Rammensee , P. Thibault , C. Perreault , M. Bassani‐Sternberg , R. Aebersold , E. Caron , Nucleic. Acids. Res. 2018, 46, D1237. 28985418 10.1093/nar/gkx664 PMC5753376

[136] W. Shao , E. Caron , P. Pedrioli , R. Aebersold , Methods Mol. Biol. 2020, 2120, 173. 32124319 10.1007/978-1-0716-0327-7_12

[137] B. Bulik‐Sullivan , J. Busby , C. D. Palmer , M. J. Davis , T. Murphy , A. Clark , M. Busby , F. Duke , A. Yang , L. Young , N. C. Ojo , K. Caldwell , J. Abhyankar , T. Boucher , M. G. Hart , V. Makarov , V. T. De Montpreville , O. Mercier , T. A. Chan , G. Scagliotti , P. Bironzo , S. Novello , N. Karachaliou , R. Rosell , I. Anderson , N. Gabrail , J. Hrom , C. Limvarapuss , K. Choquette , A. Spira , R. Rousseau , C. Voong , N. A. Rizvi , E. Fadel , M. Frattini , K. Jooss , M. Skoberne , J. Francis , R. Yelensky , Nat. Biotechnol. 2018, 37, 55. 10.1038/nbt.4313 30556813

[138] B. Chen , M. S. Khodadoust , N. Olsson , L. E. Wagar , E. Fast , C. L. Liu , Y. Muftuoglu , B. J. Sworder , M. Diehn , R. Levy , M. M. Davis , J. E. Elias , R. B. Altman , A. A. Alizadeh , Nat. Biotechnol. 2019, 37, 1332. 31611695 10.1038/s41587-019-0280-2 PMC7075463

[139] J. G. Abelin , D. Harjanto , M. Malloy , P. Suri , T. Colson , S. P. Goulding , A. L. Creech , L. R. Serrano , G. Nasir , Y. Nasrullah , C. D. McGann , D. Velez , Y. S. Ting , A. Poran , D. A. Rothenberg , S. Chhangawala , A. Rubinsteyn , J. Hammerbacher , R. B. Gaynor , E. F. Fritsch , J. Greshock , R. C. Oslund , D. Barthelme , T. A. Addona , C. M. Arieta , M. S. Rooney , Immunity 2019, 51, 766 e717. 31495665 10.1016/j.immuni.2019.08.012

[140] W. Zhao , X. Sher , PLoS Comput. Biol. 2018, 14, e1006457. 30408041 10.1371/journal.pcbi.1006457 PMC6224037

[141] J. Jin , Z. Liu , A. Nasiri , Y. Cui , S. Louis , A. Zhang , Y. Zhao , J. Hu , *bioRxiv* 2019 10.1101/830737

[142] Y. Han , D. Kim , BMC Bioinformatics 2017, 18, 585. 29281985 10.1186/s12859-017-1997-x PMC5745637

[143] Y. S. Vang , X. Xie , Bioinformatics 2017, 33, 2658. 28444127 10.1093/bioinformatics/btx264

[144] J. Hu , Z. Liu , *bioRxiv* 2017 10.1101/239236

[145] Z. Liu , Y. Cui , Z. Xiong , A. Nasiri , A. Zhang , J. Hu , Sci. Rep. 2019, 9, 794. 30692623 10.1038/s41598-018-37214-1 PMC6349913

[146] P. Phloyphisut , N. Pornputtapong , S. Sriswasdi , E. Chuangsuwanich , BMC Bioinformatics 2019, 20, 270. 31138107 10.1186/s12859-019-2892-4 PMC6540523

[147] T. J. O'Donnell , A. Rubinsteyn , M. Bonsack , A. B. Riemer , U. Laserson , J. Hammerbacher , Cell. Syst. 2018, 7, 129 e124. 29960884 10.1016/j.cels.2018.05.014

[148] J. Wu , W. Wang , J. Zhang , B. Zhou , W. Zhao , Z. Su , X. Gu , J. Wu , Z. Zhou , S. Chen , Front. Immunol. 2019, 10, 2559. 31736974 10.3389/fimmu.2019.02559 PMC6838785

[149] Y. Hu , Z. Wang , H. Hu , F. Wan , L. Chen , Y. Xiong , X. Wang , D. Zhao , W. Huang , J. Zeng , Bioinformatics 2019, 35, 4946. 31120490 10.1093/bioinformatics/btz427

[150] T. Zhao , L. Cheng , T. Zang , Y. Hu , Front. Genet. 2019, 10, 1191. 31850062 10.3389/fgene.2019.01191 PMC6892951

[151] K. Kim , H. S. Kim , J. Y. Kim , H. Jung , J.‐M. Sun , J. S. Ahn , M.‐J. Ahn , K. Park , S.‐H. Lee , J. K. Choi , Nat. Commun. 2020, 11, 951. 32075964 10.1038/s41467-020-14562-z PMC7031381

[152] H. Zeng , D. K. Gifford , Bioinformatics 2019, 35, i278. 31510651 10.1093/bioinformatics/btz330 PMC6612839

[153] X. Xie , Y. Han , K. Zhang , 2019 IEEE Int. Conf. on Bioinformatics and Biomedicine (BIBM), IEEE, Piscataway, NJ 2019, pp. 548–554.

[154] Z. Liu , J. Jin , Y. Cui , Z. Xiong , A. Nasiri , Y. Zhao , J. Hu , *bioRxiv* 2019 10.1101/817502

[155] J.‐W. Sidhom , D. Pardoll , A. Baras , *bioRxiv* 2018 10.1101/318881

[156] X. M. Shao , R. Bhattacharya , J. Huang , I. K. A. Sivakumar , C. Tokheim , L. Zheng , D. Hirsch , B. Kaminow , A. Omdahl , M. Bonsack , A. B. Riemer , V. E. Velculescu , V. Anagnostou , K. A. Pagel , R. Karchin , Cancer Immunol. Res. 2020, 8, 396. 31871119 10.1158/2326-6066.CIR-19-0464 PMC7056596

[157] H. Zeng , D. K. Gifford , Cell. Syst. 2019, 9, 159 e153. 31176619 10.1016/j.cels.2019.05.004 PMC6715517

[158] J. Vielhaben , M. Wenzel , W. Samek , N. Strodthoff , BMC Bioinformatics 2020, 21, 279. 32615972 10.1186/s12859-020-03631-1 PMC7330990

[159] J. S. Blum , P. A. Wearsch , P. Cresswell , Annu. Rev. Immunol. 2013, 31, 443. 23298205 10.1146/annurev-immunol-032712-095910 PMC4026165

[160] M. E. Peters , M. Neumann , M. Iyyer , M. Gardner , C. Clark , K. Lee , L. Zettlemoyer , arXiv preprint, arXiv:1802.05365, 2018.

[161] S. Sarkizova , S. Klaeger , P. M. Le , L. W. Li , G. Oliveira , H. Keshishian , C. R. Hartigan , W. Zhang , D. A. Braun , K. L. Ligon , P. Bachireddy , I. K. Zervantonakis , J. M. Rosenbluth , T. Ouspenskaia , T. Law , S. Justesen , J. Stevens , W. J. Lane , T. Eisenhaure , G. L. Zhang , K. R. Clauser , N. Hacohen , S. A. Carr , C. J. Wu , D. B. Keskin , Nat. Biotechnol. 2020, 38, 199. 31844290 10.1038/s41587-019-0322-9 PMC7008090

[162] B. Kuhlman , P. Bradley , Nat. Rev. Mol. Cell. Biol. 2019, 20, 681. 31417196 10.1038/s41580-019-0163-x PMC7032036

[163] M. Grabowski , E. Niedzialkowska , M. D. Zimmerman , W. Minor , J. Struct. Funct. Genomics. 2016, 17, 1. 26935210 10.1007/s10969-016-9201-5 PMC4834271

[164] W. Kabsch , C. Sander , Biopolymers 1983, 22, 2577. 6667333 10.1002/bip.360221211

[165] W. Wardah , M. G. M. Khan , A. Sharma , M. A. Rashid , Comput. Biol. Chem. 2019, 81, 1. 31442779 10.1016/j.compbiolchem.2019.107093

[166] B. Zhang , J. Li , Q. Lu , BMC Bioinformatics 2018, 19, 293. 30075707 10.1186/s12859-018-2280-5 PMC6090794

[167] C. Fang , Y. Shang , D. Xu , Proteins 2018, 86, 592. 29492997 10.1002/prot.25487 PMC6120586

[168] R. Heffernan , Y. Yang , K. Paliwal , Y. Zhou , Bioinformatics 2017, 33, 2842. 28430949 10.1093/bioinformatics/btx218

[169] Y. Guo , B. Wang , W. Li , B. Yang , J. Bioinform. Comput. Biol. 2018, 16, 1850021. 30419785 10.1142/S021972001850021X

[170] Y. Guo , W. Li , B. Wang , H. Liu , D. Zhou , BMC Bioinformatics 2019, 20, 341. 31208331 10.1186/s12859-019-2940-0 PMC6580607

[171] S. Wang , J. Peng , J. Ma , J. Xu , Sci. Rep. 2016, 6, 18962. 26752681 10.1038/srep18962 PMC4707437

[172] R. Das , D. Baker , Annu. Rev. Biochem. 2008, 77, 363. 18410248 10.1146/annurev.biochem.77.062906.171838

[173] S. Ovchinnikov , L. Kinch , H. Park , Y. Liao , J. Pei , D. E. Kim , H. Kamisetty , N. V. Grishin , D. Baker , eLife 2015, 4, e09248. 26335199 10.7554/eLife.09248 PMC4602095

[174] D. S. Marks , L. J. Colwell , R. Sheridan , T. A. Hopf , A. Pagnani , R. Zecchina , C. Sander , PLoS One 2011, 6, e28766. 22163331 10.1371/journal.pone.0028766 PMC3233603

[175] L. A. Abriata , G. E. Tamo , M. Dal Peraro , Proteins 2019, 87, 1100. 31344267 10.1002/prot.25787

[176] J. Moult , K. Fidelis , A. Kryshtafovych , T. Schwede , A. Tramontano , Proteins 2016, 84, 4.27171127

[177] F. Morcos , A. Pagnani , B. Lunt , A. Bertolino , D. S. Marks , C. Sander , R. Zecchina , J. N. Onuchic , T. Hwa , M. Weigt , Proc. Natl. Acad. Sci. U S A. 2011, 108, E1293. 22106262 10.1073/pnas.1111471108 PMC3241805

[178] H. Kamisetty , S. Ovchinnikov , D. Baker , Proc. Natl. Acad. Sci. U S A. 2013, 110, 15674. 24009338 10.1073/pnas.1314045110 PMC3785744

[179] D. T. Jones , D. W. Buchan , D. Cozzetto , M. Pontil , Bioinformatics 2012, 28, 184. 22101153 10.1093/bioinformatics/btr638

[180] D. T. Jones , S. M. Kandathil , Bioinformatics 2018, 34, 3308. 29718112 10.1093/bioinformatics/bty341 PMC6157083

[181] B. Adhikari , J. Hou , J. Cheng , Bioinformatics 2018, 34, 1466. 29228185 10.1093/bioinformatics/btx781 PMC5925776

[182] S. Wang , S. Sun , Z. Li , R. Zhang , J. Xu , PLoS Comput. Biol. 2017, 13, e1005324. 28056090 10.1371/journal.pcbi.1005324 PMC5249242

[183] M. AlQuraishi , Bioinformatics 2019, 35, 4862. 31116374 10.1093/bioinformatics/btz422 PMC6907002

[184] A. W. Senior , R. Evans , J. Jumper , J. Kirkpatrick , L. Sifre , T. Green , C. Qin , A. Žídek , A. W. R. Nelson , A. Bridgland , H. Penedones , S. Petersen , K. Simonyan , S. Crossan , P. Kohli , D. T. Jones , D. Silver , K. Kavukcuoglu , D. Hassabis , Nature 2020, 577, 706. 31942072 10.1038/s41586-019-1923-7

[185] A. W. Senior , R. Evans , J. Jumper , J. Kirkpatrick , L. Sifre , T. Green , C. Qin , A. Žídek , A. W. R. Nelson , A. Bridgland , H. Penedones , S. Petersen , K. Simonyan , S. Crossan , P. Kohli , D. T. Jones , D. Silver , K. Kavukcuoglu , D. Hassabis , Proteins 2019, 87, 1141. 31602685 10.1002/prot.25834 PMC7079254

[186] K. T. Simons , C. Kooperberg , E. Huang , D. Baker , J. Mol. Biol. 1997, 268, 209. 9149153 10.1006/jmbi.1997.0959

[187] J. Xu , S. Wang , Proteins 2019, 87, 1069. 31471916 10.1002/prot.25810

[188] R. Shrestha , E. Fajardo , N. Gil , K. Fidelis , A. Kryshtafovych , B. Monastyrskyy , A. Fiser , Proteins 2019, 87, 1058. 31587357 10.1002/prot.25819 PMC6851495

[189] A. T. Brunger , Nat. Protoc. 2007, 2, 2728. 18007608 10.1038/nprot.2007.406

[190] W. Zheng , Y. Li , C. Zhang , R. Pearce , S. M. Mortuza , Y. Zhang , Proteins 2019, 87, 1149. 31365149 10.1002/prot.25792 PMC6851476

[191] J. Hou , T. Wu , R. Cao , J. Cheng , Proteins 2019, 87, 1165. 30985027 10.1002/prot.25697 PMC6800999

[192] T. I. Croll , M. D. Sammito , A. Kryshtafovych , R. J. Read , Proteins 2019, 87, 1113. 31407380 10.1002/prot.25800 PMC6851432

[193] B. Adhikari , D. Bhattacharya , R. Cao , J. Cheng , Proteins 2015, 83, 1436. 25974172 10.1002/prot.24829 PMC4509844

[194] H. Fukuda , K. Tomii , BMC Bioinformatics 2020, 21, 10. 31918654 10.1186/s12859-019-3190-x PMC6953294

[195] M. Gao , H. Zhou , J. Skolnick , Sci. Rep. 2019, 9, 3514. 30837676 10.1038/s41598-019-40314-1 PMC6401133

[196] J. Yang , I. Anishchenko , H. Park , Z. Peng , S. Ovchinnikov , D. Baker , Proc. Natl. Acad. Sci. U S A. 2020, 117, 1496. 31896580 10.1073/pnas.1914677117 PMC6983395

[197] C. Zhang , W. Zheng , S. M. Mortuza , Y. Li , Y. Zhang , Bioinformatics 2020, 36, 2105. 31738385 10.1093/bioinformatics/btz863 PMC7141871

[198] S. M. Kandathil , J. G. Greener , D. T. Jones , Proteins 2019, 87, 1179. 31589782 10.1002/prot.25824 PMC6899861

[199] M. AlQuraishi , Cell. Syst. 2019, 8, 292 e293. 31005579 10.1016/j.cels.2019.03.006 PMC6513320

[200] J. Ingraham , A. J. Riesselman , C. Sander , D. S. Marks , presented at ICLR 2019 Conf., New Orleans, LA, May 2019.

[201] J. J. Almagro Armenteros , C. K. Sonderby , S. K. Sonderby , H. Nielsen , O. Winther , Bioinformatics 2017, 33, 3387. 29036616 10.1093/bioinformatics/btx431

[202] W. Long , Y. Yang , H. B. Shen , Bioinformatics 2020, 36, 2244. 31804670 10.1093/bioinformatics/btz909

[203] T. Sun , B. Zhou , L. Lai , J. Pei , BMC Bioinformatics 2017, 18, 277. 28545462 10.1186/s12859-017-1700-2 PMC5445391

[204] S. Hashemifar , B. Neyshabur , A. A. Khan , J. Xu , Bioinformatics 2018, 34, i802. 30423091 10.1093/bioinformatics/bty573 PMC6129267

[205] M. Kulmanov , R. Hoehndorf , Bioinformatics 2020, 36, 422. 31350877 10.1093/bioinformatics/btz595 PMC9883727

[206] A. Sureyya Rifaioglu , T. Dogan , M. Jesus Martin , R. Cetin‐Atalay , V. Atalay , Sci. Rep. 2019, 9, 7344. 31089211 10.1038/s41598-019-43708-3 PMC6517386

[207] G. Zhou , M. Chen , C. J. T. Ju , Z. Wang , J.‐Y. Jiang , W. Wang , NAR Genom. Bioinform. 2020, 2, lqaa015. 32166223 10.1093/nargab/lqaa015 PMC7059401

[208] H. Y. Kim , D. Kim , Bioinformatics 2020, 36, 2047. 31746978 10.1093/bioinformatics/btz873

[209] R. Dehghanpoor , E. Ricks , K. Hursh , S. Gunderson , R. Farhoodi , N. Haspel , B. Hutchinson , F. Jagodzinski , Molecules 2018, 23, 251. 10.3390/molecules23020251 PMC6017198 29382060

[210] G. Serrano , E. Guruceaga , V. Segura , Bioinformatics 2020, 36, 1279. 31529040 10.1093/bioinformatics/btz708

[211] H. Kim , Y. Kim , B. Han , J. Y. Jang , Y. Kim , J. Proteome. Res. 2019, 18, 3195. 31314536 10.1021/acs.jproteome.9b00268

[212] H. Dong , Y. Liu , W. F. Zeng , K. Shu , Y. Zhu , C. Chang , Proteomics 2020, e1900344. 32643271 10.1002/pmic.201900344

[213] F. Zhang , S. Yu , L. Wu , Z. Zang , X. Yi , J. Zhu , C. Lu , P. Sun , Y. Sun , S. Selvarajan , L. Chen , X. Teng , Y. Zhao , G. Wang , J. Xiao , S. Huang , O. L. Kon , N. G. Iyer , S. Z. Li , Z. Luan , T. Guo , bioRxiv 2020 10.1101/2020.03.05.978635

[214] C. Escher , L. Reiter , B. MacLean , R. Ossola , F. Herzog , J. Chilton , M. J. MacCoss , O. Rinner , Proteomics 2012, 12, 1111. 22577012 10.1002/pmic.201100463 PMC3918884

[215] Y. Perez‐Riverol , A. Csordas , J. Bai , M. Bernal‐Llinares , S. Hewapathirana , D. J. Kundu , A. Inuganti , J. Griss , G. Mayer , M. Eisenacher , E. Pérez , J. Uszkoreit , J. Pfeuffer , T. Sachsenberg , S. Yilmaz , S. Tiwary , J. Cox , E. Audain , M. Walzer , A. F. Jarnuczak , T. Ternent , A. Brazma , J. A. Vizcaíno , Nucleic. Acids. Res. 2019, 47, D442. 30395289 10.1093/nar/gky1106 PMC6323896

[216] E. W. Deutsch , N. Bandeira , V. Sharma , Y. Perez‐Riverol , J. J. Carver , D. J. Kundu , D. García‐Seisdedos , A. F. Jarnuczak , S. Hewapathirana , B. S. Pullman , J. Wertz , Z. Sun , S. Kawano , S. Okuda , Y. Watanabe , H. Hermjakob , B. MacLean , M. J. MacCoss , Y. Zhu , Y. Ishihama , J. A. Vizcaíno , Nucleic. Acids. Res. 2020, 48, D1145. 31686107 10.1093/nar/gkz984 PMC7145525

[217] A. Vaswani , N. Shazeer , N. Parmar , J. Uszkoreit , L. Jones , A. N. Gomez , Ł. Kaiser , I. Polosukhin , Advances in Neural Information Processing Systems, Curran Associates, Inc., New York 2017, pp. 5998–6008.

[218] J. Devlin , M.‐W. Chang , K. Lee , K. Toutanova , arXiv preprint, arXiv:1810.04805, 2018.

[219] A. Radford , J. Wu , R. Child , D. Luan , D. Amodei , I. Sutskever , OpenAI Blog 2019, 1, 9.

[220] A. Nambiar , M. E. Heflin , S. Liu , S. Maslov , A. Ritz , BioRxiv 2020 10.1101/2020.06.15.153643

[221] L. Chen , X. Tan , D. Wang , F. Zhong , X. Liu , T. Yang , X. Luo , K. Chen , H. Jiang , M. Zheng , Bioinformatics 2020, btaa524, 10.1093/bioinformatics/btaa524 32428219

[222] Z. Wu , S. Pan , F. Chen , G. Long , C. Zhang , P. S. Yu , IEEE Trans. Neural. Netw. Learn. Syst. 2020, 1, 10.1109/TNNLS.2020.2978386. 32217482

[223] A. Fout , J. Byrd , B. Shariat , A. Ben‐Hur , Advances in Neural Information Processing Systems, Curran Associates, Inc., New York 2017, pp. 6530–6539.

[224] Z. Chen , X. Liu , F. Li , C. Li , T. Marquez‐Lago , A. Leier , T. Akutsu , G. I. Webb , D. Xu , A. I. Smith , L. Li , K.‐C. Chou , J. Song , Brief. Bioinform. 2019, 20, 2267. 30285084 10.1093/bib/bby089 PMC6954452

